# Therapeutic hypothermia in neonatal hypoxic encephalopathy: A systematic review and meta-analysis

**DOI:** 10.7189/jogh.12.04030

**Published:** 2022-04-09

**Authors:** Joseph L Mathew, Navneet Kaur, Jeanne M Dsouza

**Affiliations:** 1 Advanced Pediatrics Centre, Postgraduate Institute of Medical Education and Research Chandigarh, India; 2 Kasturba Medical College, Manipal, India

## Abstract

**Background:**

Therapeutic hypothermia (TH) is regarded as the most efficacious therapy for neonatal hypoxic encephalopathy. However, limitations in previous systematic reviews and the publication of new data necessitate updating the evidence. We conducted this up-to-date systematic review to evaluate the effects of TH in neonatal encephalopathy on clinical outcomes.

**Methods:**

In this systematic review and meta-analysis, we searched Medline, Cochrane Library, Embase, LIVIVO, Web of Science, Scopus, CINAHL, major trial registries, and grey literature (from inception to October 31, 2021), for randomized controlled trials (RCT) comparing TH vs normothermia in neonatal encephalopathy. We included RCTs enrolling neonates (gestation ≥35 weeks) with perinatal asphyxia and encephalopathy, who received either TH (temperature ≤34°C) initiated within 6 hours of birth for ≥48 hours, vs no cooling. We excluded non-RCTs, those with delayed cooling, or cooling to >34°C. Two authors independently appraised risk-of-bias and extracted data on mortality and neurologic disability at four time points: neonatal (from randomization to discharge/death), infancy (18-24 months), childhood (5-10 years), and long-term (>10 years). Other outcomes included seizures, EEG abnormalities, and MRI findings. Summary data from published RCTs were pooled through fixed-effect meta-analysis.

**Results:**

We identified 36 863 citations and included 39 publications representing 29 RCTs with 2926 participants. Thirteen studies each had low, moderate, and high risk-of-bias. The pooled risk ratios (95% confidence interval, CI) were as follows: neonatal mortality: 0.87 (95% CI = 0.75, 1.00), n = 2434, *I^2^* = 38%; mortality at 18-24 months: 0.88 (95% CI = 0.78, 1.01), n = 2042, *I^2^* = 51%; mortality at 5-10 years: 0.81 (95% CI = 0.62, 1.04), n = 515, *I^2^* = 59%; disability at 18-24 months: 0.62 (95% CI = 0.52, 0.75), n = 1440, *I^2^* = 26%; disability at 5-10 years: 0.68 (95% CI = 0.52, 0.90), n = 442, *I^2^* = 3%; mortality or disability at 18-24 months: 0.78 (95% CI = 0.72, 0.86), n = 1914, *I^2^* = 54%; cerebral palsy at 18-24 months: 0.63 (95% CI = 0.50, 0.78), n = 1136, *I^2^* = 39%; and childhood cerebral palsy: 0.63 (95% CI = 0.46, 0.85), n = 449, *I^2^* = 0%. Some outcomes showed significant differences by study-setting; the risk ratio (95% CI) for mortality at 18-24 months was 0.79 (95% CI = 0.66,0.93), n = 1212, *I^2^* = 7% in high-income countries, 0.67 (95% CI = 0.41, 1.09), n = 276, *I^2^* = 0% in upper-middle-income countries, and 1.18 (95% CI = 0.94, 1.47), n = 554, *I^2^* = 75% in lower-middle-income countries. The corresponding pooled risk ratios for ‘mortality or disability at 18-24 months’ were 0.77 (95% CI = 0.69, 0.86), n = 1089, *I^2^* = 0%; 0.56 (95% CI = 0.41, 0.78), n = 276, *I^2^* = 30%; and 0.92 (95% CI = 0.77, 1.09), n = 549, *I^2^* = 86% respectively. Trials with low risk of bias showed risk ratio of 0.97 (95% CI = 0.80, 1.16, n = 1475, *I^2^* = 62%) for neonatal mortality, whereas trials with higher risk of bias showed 0.71 (95% CI = 0.55, 0.91), n = 959, *I^2^* = 0%. Likewise, risk ratio for mortality at 18-24 months was 0.96 (95% CI = 0.83, 1.13), n = 1336, *I^2^* = 58% among low risk-of-bias trials, but 0.72 (95% CI = 0.56, 0.92), n = 706, *I^2^* = 0%, among higher risk of bias trials.

**Conclusions:**

Therapeutic hypothermia for neonatal encephalopathy reduces neurologic disability and cerebral palsy, but its effect on neonatal, infantile and childhood mortality is uncertain. The setting where it is implemented affects the outcomes. Low(er) quality trials overestimated the potential benefit of TH.

Neonatal hypoxic ischemic encephalopathy is a significant cause of mortality and morbidity. It is also associated with adverse outcomes such as cerebral palsy, cognitive dysfunction, epilepsy, and others, well beyond the neonatal period. These have a cascading impact on the community and society through increased health care utilization, need for special services, economic burden, and diminished workforce productivity. Several interventions have been explored to manage neonatal encephalopathy (NE). Among these, therapeutic hypothermia (TH) is ranked highest, with several studies and systematic reviews [1,2] reporting reduction in mortality and adverse neurological and/or neurodevelopmental outcomes during infancy [3,4]. TH involves controlled cooling of the body (or at least of the head) during the first 2-4 days of life, followed by a gradual rewarming to a euthermic state [1,5]. Currently, it is implemented globally, including in many low-resource health care settings [6-8], although the International Liaison Committee on Resuscitation advised its use only in institutions with adequate monitoring and intensive care facilities [9].

A recent multi-country HELIX trial reported that TH was associated with an alarming increase in both immediate and late mortality, prompting the authors to emphatically recommend its immediate discontinuation in resource-constrained settings [10]. This created considerable consternation, especially in some developing countries, with arguments about the trial methods, generalizability, and other issues [11-17]. However, critical appraisal of the trial confirmed its validity [18], despite some plausible explanations for the stark differences in key outcomes [19]. Additionally, a systematic review restricted to trials from developing countries reported limited benefit of TH in such settings [20].

These developments necessitate a detailed review of the available evidence. The Cochrane review published in 2013 is outdated, and also contained some data analysis errors, such as combining short-term and long-term outcomes in the same meta-analysis [1]. A more recent review, updated as of mid-2020, contained several errors such as duplication of data from some trials, presenting data from non-existent trials, missing relevant trials, combining short-term and long-term mortality together, and expressing relative risk with negative integers [2]. Therefore, we conducted an up-to-date systematic review of randomized controlled trials (RCTs) to evaluate the effects of therapeutic hypothermia (Intervention), vs normothermia or no hypothermia (Comparison), in neonates with hypoxic encephalopathy (Population), on mortality and neurological and/or neurodevelopmental features (Outcomes). The question of this review was: What are the effects of therapeutic hypothermia in newborns with hypoxic encephalopathy?

## METHODS

This review was registered in PROSPERO (Registration number CRD42021279682, dated 20 October 2021) [21] and conducted in accordance with the Cochrane Handbook for systematic reviews [22]. The review is reported according to the Preferred Reporting Items for Systematic Reviews and Meta-Analyses-Protocols (PRISMA-P) 2020 statement [23].

### Criteria for considering studies for this review

**Types of studies:** We included RCTs comparing the use of therapeutic hypothermia vs normothermia, or no hypothermia. We excluded non-randomized trials, cohort studies, trials with historic controls, case series, trials in animals, in vitro experiments, and ex vivo human studies.

**Types of participants:** We included RCTs enrolling newborn infants with a gestational age ≥35 weeks, having evidence of perinatal asphyxia and encephalopathy. Perinatal asphyxia was defined by one or more of the following: a) Apgar score ≤5 at 5 minutes of life; b) need for ongoing resuscitation or respiratory support at 10 minutes; or c) cord blood/arterial blood pH<7.1, or base deficit ≥12 within one hour of birth. Evidence of encephalopathy was based on Sarnat staging system or any other recognized staging/classification system.

**Types of intervention:** We included RCTs delivering TH (whole-body cooling [WBC] or selective head cooling [SHC]) by any device/equipment, initiated within 6 hours of birth, with documented reduction in core temperature (to ≤34°C in case of WBC) or middle ear temperature (to ≤34°C in case of SHC). We excluded trials where TH was initiated later than six hours after birth (in all or the majority of infants), or cooling was conducted without documentation of core temperature (as specified above), or was done for <48 hours.

**Types of comparison:** The comparator was normothermia, or no therapeutic cooling, or no intervention. We excluded studies without a comparison group, those in which the comparison group had received any cooling for any duration, or a historic comparison group.

**Types of outcome measures:** We considered the following outcomes: mortality, neurological impairment or disability (defined by any standard criteria), the composite outcome of mortality or disability, and cerebral palsy. We assessed these at four time points after randomization: a) Neonatal, ie, from randomization to discharge or death during the initial hospitalization; b) Infancy, ie, at the age of 18-24 months, c) Childhood, ie, at the age of 5-10 years, and d) Long-term, ie, beyond the age of 10 years. Other outcomes were seizures, electroencephalogram (aEEG) abnormalities, MRI findings suggesting neuronal damage during the initial hospitalization, duration of hospitalization, and quality of life. For this analysis, the primary outcome was listed as “mortality or neurological disability” at ≥18 months of age [21].

**Information sources:** Two authors independently searched the following databases: Medline, Embase, Cochrane Library, LIVIVO, Web of Science, Scopus, and CINAHL. We searched the following clinical trial registries: World Health Organization International Clinical Trials Registry Platform, ClinicalTrials.gov, and Clinical Trials Registry – India. We also hand-searched reference lists of included trials, as well as previous (narrative and systematic) reviews. In addition, we conducted a grey literature search using OpenGrey (www.opengrey.eu/), ProQuest, and Google Scholar. Each database was searched from its date of inception to October 31, 2021, without restrictions based on language or geography.

**Search strategy:** We used combinations of MeSH terms and synonyms of the following keywords, and their variations: neonate, newborn, perinatal, infant, hypothermia, therapeutic hypothermia, cool, cooling, therapeutic cooling, asphyxia, hypoxia, hypoxic-ischemic, encephalopathy, neonatal encephalopathy. The searches were pilot-tested before finalizing the strategy. The search strategy in representative databases is summarized in Table S1 in the **Online Supplementary Document**.

**Selection of studies:** Two review authors independently screened citation titles, followed by the abstracts of short-listed citations, followed by full-text of potentially eligible studies (and those without abstracts). Thereafter, two authors independently examined the full text versions of short-listed studies, to confirm eligibility for inclusion, and recorded reasons for exclusion of ineligible studies. Disagreements were discussed and resolved by consensus. After eliminating duplicate publications, a final list of studies was prepared. A PRISMA flow diagram was created, summarizing the search results and process of including studies.

**Translation of languages other than English**: Non-English publication abstracts were translated using open-source software; if eligible, the full text was translated as well.

**Data extraction:** Two review authors independently extracted the following information from the included studies.

- Trial characteristics: design, study duration, setting, date of publication.
- Participant characteristics: inclusion criteria, exclusion criteria, gestational age, birth weight, definition of perinatal asphyxia, definition and severity of encephalopathy, sample size.
- Intervention characteristics: WBC or SHC, method of cooling, temperature targeted, method of determining target temperature, cooling duration, cooling cessation criteria.
- Comparison characteristics: Temperature targeted, method of determining target temperature, and standard of care.
- Outcomes: Data on the outcomes listed above were extracted along with notes/remarks.

**Dealing with missing data:** We attempted to contact the corresponding authors of studies with missing or unclear data.

**Data synthesis and statistical analysis**: We presented data on baseline characteristics with descriptive statistics. We pooled data on the outcomes of interest and performed meta-analysis, using Cochrane Review Manager version 5.4 [24]. For dichotomous outcomes, we calculated risk ratios (RR) with 95% confidence interval (CI) using the fixed-effect model. For continuous outcomes, we calculated the weighted mean difference with 95% CI (fixed-effect model). We opted for the fixed-effect model, as the alternative (random effects-model) tends to assign disproportionately greater weight to studies with smaller sample sizes. However, wherever the heterogeneity statistic exceeded 50%, we re-examined the pooled effect with the random effects model also. For data that could not be pooled by meta-analysis, we provided a description, summarizing the key results.

**Assessment of methodological quality of included studies:** Two authors independently assessed methodological quality, using version 2 of the Cochrane Risk-of-Bias (RoB) tool [25]. We assessed RoB for each reported outcome of each trial, and the overall RoB of each trial.

**Assessment of heterogeneity:** We assessed heterogeneity among trials by visual inspection of the forest plots, and the Higgins-Thompson I^2^ method. We interpreted heterogeneity as outlined in the Cochrane Handbook: 75%-100% = considerable heterogeneity, 50%-90% = may represent substantial heterogeneity, 30%-60% = may represent moderate heterogeneity, and 0%-40% = might not be important [22]. Where *I^2^* exceeded 50%, we tried to identify explanations.

**Subgroup analysis:** We conducted a subgroup analysis based on the following criteria: a) Study setting (defined by the World Bank Classification of the country where the trial was conducted): high-income country (HIC), upper middle-income country (UMIC), lower middle-income country (LMIC), low-income country (LIC); and b) Type of cooling: WBC vs SHC. We planned subgroup analysis based on cooling method (formal devices vs informal methods), but there were insufficient studies.

**Sensitivity analysis**: We assessed the impact of low(er) quality studies, by excluding trials with moderate/high RoB.

## RESULTS

**Figure 1:**
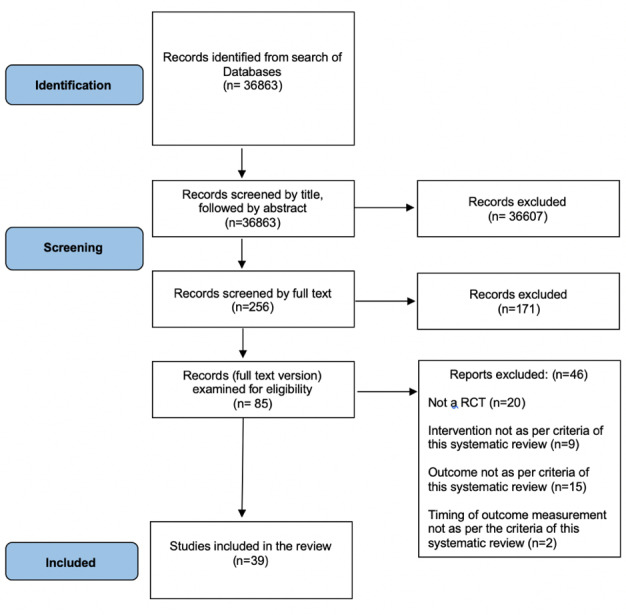
Flowchart highlighting screening and selection of studies.

We identified 36 863 citations, of which 85 citations were short-listed, and 39 publications [10,26-63] reporting 29 trials with 2926 participants [10,26-32,34-36,38,39,41-47,49,50,53,57-62] were included (**Figure 1**). Characteristics of the included studies are presented in **Table 1**, and their detailed description in Table S2 in the **Online Supplementary Document**. The reasons for excluding 46 studies [64-109] are presented in Table S3 in the **Online Supplementary Document**. Two authors independently categorized 13 studies each as having overall high, moderate, and low RoB (Table S4 in the **Online Supplementary Document**).

**Table 1:** Characteristics of the included studies

No.	Trials (n = 29)	Publications (n = 39)	Country	Inclusion criteria	Exclusion criteria	Number randomized		Type of cooling and method	Site of temperature measurement	Target temperature (°C)	
**TH**	**Control**	**TH**	**Control**
1	Akisu	Akisu 2003 [32]	Turkey	5 min AS<6; cord blood or arterial pH 7.1 or BD>10 mmol/l; encephalopathy (stupor, hypotonia, abnormal neonatal reflexes)	Metabolic disorders, congenital malformations, chromosomal abnormalities, congenital infection, transitory drug depression	11	10	SHC (cooling caps)	EAC; rectal	33.0-33.5	36.0-36.5
2	Battin	Battin 2001 [34]*_,_ Battin 2003 [63]^†^	New Zealand	GA≥37 wk; 5 min AS≤6 or cord/first arterial pH≤7.09; encephalopathy (lethargy/stupor, hypotonia, abnormal reflexes)	Major congenital abnormalities	25	15	SHC (cooling caps)	rectal	36.5-36 (n = 6); 35.9-35.5 (n = 6); 35.0 ± 0.5 (n = 6); 34.5 ± 0.5 (n = 7)	37.0 ± 0.2
3	Bharadwaj	Bharadwaj 2012 [35]	India	GA>37 wk with HIE; ABG pH≤7 or BE≥-12 meq within 1st h and also fulfilling any two of, AS≤6 at 10 min; evidence of fetal distress; assisted ventilation for at least 10 min after birth; evidence of any organ dysfunction; and history of acute perinatal event with evidence of encephalopathy	Age >6 h, major congenital anomalies, if the infant did not establish spontaneous respiration by 20 min after birth, out-born babies	65	65	WBC (gel packs)	rectal	33.0-34.0	36.5
4	Bhat	Bhat 2006 [36]	India	NA	NA	20	15	WBC (not mentioned)	rectal, skin	33.5	NA
5	Catherine	Catherine 2020 [38]	India	pH≤7 or BD≤12 meq in cord blood; AS≤6 at 10 min, any clinical evidence of fetal distress, requiring assisted ventilation for at least 10 min after delivery, and any evidence of one or more organ dysfunction	Age >6 h, out-born babies, major congenital abnormalities, no spontaneous respiratory efforts by 20 min following delivery	78	84	WBC (phase changing material)	rectal	33.5 ± 0.5	36.5
6	Chen	Chen 2018 [39]	China	NA	NA	20	20	SHC (not mentioned)	rectal	34.5-35.0	NA
7	CoolCap Trial	Gluckman 2005 [29]	USA	AS≤5 at 10 min after birth; need for resuscitation at 10 min after birth; or severe acidosis (pH<7.00 or BD≥16 mmol/l in cord, arterial or venous sample within 60min of birth)	Age >5.5 h, prophylactic high-dose anticonvulsants, major congenital abnormalities, head trauma causing major intracranial hemorrhage, severe growth restriction, BW<1800 g, infants judged critically ill, unavailability of essential equipment, planned concurrent participation in other experimental treatments	116	118	SHC (cooling caps)	rectal	34.0-35.0	36.8-37.2
8	Eicher	Eicher 2005 [41]	USA	GA≥35 wk, BW 2000 g, hypoxic-ischemic insult, with one clinical sign & two neurologic findings of hypoxia-ischemia, cord pH 7.0 or BD 13, initial infant pH 7.1, AS 5 at 10 min, continued resuscitation after 5 min, fetal bradycardia with HR 80/min lasting 15 min, or postnatal hypoxic ischemic event with oxygen desaturation 70% or arterial oxygen tension 35 mm Hg for 20 min with evidence of ischemia	Clinical sepsis, maternal chorioamnionitis, weight or head circumference <10th percentile for gestation age, or congenital abnormalities	32	33	WBC (cooling blanket)	rectal	33.0 ± 0.5	37.0 ± 0.5
9	El Shimi	El Shimi 2014 [57]	Egypt	pH≤7.0 or BD≥16 mmol/l in cord or any blood during 1st h after birth. If pH 7.01-7.15, BD 10.0-15.9 mmol/l, or blood gas unavailable, additional criteria viz. acute perinatal event (late or variable decelerations, cord prolapse, cord rupture, uterine rupture, maternal trauma, hemorrhage, or cardiorespiratory arrest) and either 10 min AS≤5 or assisted ventilation initiated at birth and continued for >10 min	Major congenital anomalies, severe growth restriction (BW 1800g), presence of an infectious cause, suspected inborn error of metabolism, age >6 h	10	10	WBC (cool packs)	rectal	33.0-34.0	NA
10	HELIX trial	Thayyil 2021 [10]	India, Sri Lanka, Bangladesh	GA≥37 wk, BW≥1kg, need for resuscitation at 5 min of age or AS<6 at 5 min of age (for babies born in hospital), or both, or absence of crying by 5 min of age (for babies born at home); and evidence of moderate or severe encephalopathy between 1-6 h of age	No heart rate at 10 min of age despite adequate resuscitation, with major life-threatening congenital malformations	202	206	WBC (servo-controlled cooling device)	rectal	33.5 ± 0.10	36.7 ± 0.06
11	ICE trial	Jacobs 2011 [26], Cheong 2012 [40]	Australia, New Zealand, Canada, USA	GA≥35 wk, age <6 h, moderate or severe encephalopathy and indicators of peripartum hypoxia-ischemia (≥2 of the following, AS≤5 at 10 min, continued need for mechanical ventilation at 10 min, and/or metabolic acidosis (cord, arterial or venous pH<7.00; or BD≥12 within 60 min of birth)	Age >6 h, BW<2 kg, major congenital abnormalities, bleeding, >80% inspired oxygen, imminent death, TH before assessment	110	111	WBC (gel packs)	rectal	33.0-34.0	37.0
12	Inder	Inder 2004 [43]	Australia	GA≥35 with intrapartum hypoxia-ischemia comprising at least two of, AS≤5 at 10 min; ongoing resuscitation at 10 min; and metabolic acidosis (cord pH≤7 or BD≥12 mmol/l or more within 60 min of life) combined with clinical evidence of encephalopathy	NA	13	14	WBC (cool packs)	rectal	33.0-34.0	36.8-37.3
13	Jose	Jose 2018 [44]	India	Moderate or severe encephalopathy within 6 h after birth after an acute perinatal event, with acidosis or resuscitation	NA	77	79	WBC (not mentioned)	NA	33.0	NA
14	Joy	Joy 2012 [45]	India	GA≥37 wk with cord or peripheral blood pH≤7 or BD≥12 mEq within 1 h with evidence of encephalopathy and with any two of, AS at 10 min ≤5; assisted ventilation for at least ≥10 min after birth; evidence of any organ dysfunction; history of acute perinatal event (intrapartum fetal distress, cord prolapse, placental abruption, uterine rupture, maternal trauma, or cardiac arrest)	Age >6 h, major congenital abnormalities; no spontaneous respiration by 20 min, out-born babies	58	58	WBC (gel packs)	rectal	33.0-34.0	36.5
15	Li	Li 2009 [46]	China	GA≥37 wk; BW>2500 g, admitted to NICU within 10 h^$$^ history of asphyxia (AS at 5 min ≤5 with ABG pH<7.1 or BD>16 mmol/l within 1h birth); clinical evidence of encephalopathy	Major congenital abnormalities; head trauma; skull fracture; enrollment >10 h after birth	46	47	WBC (cooling mattress)	rectal	33.0-34.5	37.0
16	Lin	Lin 2006 [47]	China	GA≥37 wk; AS at 5 min <6 with postnatal ABG pH<7.1 or BD>15 mEq/l; signs of postpartum encephalopathy (decreased muscle tone, lethargy, coma, or seizures within 6 h after birth)	Major congenital abnormalities; prolonged hypoxemia due to severe persistent fetal circulation	32	30	SHC (cooling caps)	rectal; NP	34.5 ± 0.5 (rectal); 34.4 ± 0.5 (NP)	37.1 ± 0.5 (rectal); 36.8 ± 0.5 (NP)
17	neo.nEURO.network trial	Simbruner 2010 [58]	Germany	AS 5, or continued need for resuscitation at 10 min after birth, cord or any arterial pH 7.00 within 1 h after birth, BD 16 mmol/l, encephalopathy (lethargy, stupor, or coma and one of the following, hypotonia, abnormal reflexes, absent or weak suck), clinical seizures and abnormal standard EEG or aEEG findings	Age >5.5 h, high-dose anticonvulsant therapy, BW 1800 g or GA 36 wk, head circumference <3rd percentile, major congenital malformations with poor developmental prognosis, Imperforate anus gross hemorrhage, infant “in extremis”	64	65	WBC (cooling blanket)	rectal	33.0-34.0	36.5-37.5
18	NEST study	Field 2013 [42]	UK	Standard criteria for ECMO eligibility based on the clinical decision of the local ECMO team	Congenital diaphragmatic hernia, post-cardiac surgery, any therapeutic cooling before randomization	56	55	WBC (heat exchanger in the ECMO circuit)	rectal	34.0	37.0
19	NICHD trial	Shankaran 2005 [27],Shankaran 2008 [54], Shankaran 2012 [55], Shankaran 2012a [56],	USA	Cord or any blood pH≤7.0 or BD≥16 mmol/l during 1st h after birth. If pH 7.01-7.15, BD 10.0-15.9 mmol/l, or blood gas unavailable, additional criteria applied viz. acute perinatal event (late or variable decelerations, cord prolapse, cord rupture, uterine rupture, maternal trauma, hemorrhage, or cardiorespiratory arrest) and either 10 min AS≤5 or assisted ventilation initiated at birth and continued for at least 10 min	Age >6 h, major congenital abnormality, severe growth restriction (BW≤1800 g), refusal of consent by a parent or attending neonatologist; moribund infants	102	106	WBC (cooling blanket)	esophageal	33.5	36.0-37.0
20	Rakesh	Rakesh 2017 [49]	India	GA≥37 wk, cord or arterial blood pH≤7 or BD≥12 meq within 1st h, encephalopathy (Sarnat and Sarnat staging)	Age >6 h; major congenital abnormalities, absent spontaneous respiratory efforts by 20min after birth; out-born babies	60	60	WBC (phase) changing material)	rectal	33.0-34.0	NA
21	Robertson	Robertson 2008 [50]	Uganda	GA≥37 wk, need for resuscitation, and/or AS<6 at 5 min plus abnormal neurological assessment (>5 on Thompson score) from 30 min to 3 h after birth	Apnea or cyanosis, absent cardiac output for >10 min after birth, BW<2 kg	21	15	WBC (cooling mattress)	rectal	33.0-34.0	36.5
22	Shankaran	Shankaran 2002 [53]	USA	Cord or any blood pH within 1st h 7.0 or BD 16 mEq/l. If blood gas unavailable or pH at 1h 7.01-7.15 or BD 10.0-15.9 mEq/l, additional history of acute perinatal event and either AS = 5 at 10 min or continued need for ventilation initiated at birth and continued for at least 10 min	Age >6 h, chromosomal abnormality, major congenital anomaly, severe growth restriction (BW 1800 g), infant unlikely to survive, and parent or attending neonatologist refusal of consent	9	10	WBC (cooling blanket)	oesophageal	34.5	36.5
23	Sun	Sun 2012 [59]	China	AS<3 at 1 min and <5 at 5 min; pH<7; BD≤16 mmol/l; HIE	Major congenital abnormalities, infection on admission, severe anemia, other encephalopathy	23	28	SHC (cooling caps)	rectal	34.5-35.0	36-37.5
24	Tanigasalam	Tanigasalam 2015 [60]	India	Encephalopathy, pH 7 or BD 12meq in cord blood and fulfilling any two of, AS≤5 at 10 min of life, evidence of fetal distress, assisted ventilation for at least 10 min after birth, evidence of any organ dysfunction	Age >6 h, out-born babies, major congenital abnormalities, no spontaneous respiratory efforts by 20 min after birth or history of maternal renal failure	60	60	WBC (gel packs)	rectal	33.0-34.0	36.5
25	Thayyil	Thayyil 2013 [28]	India	Age ≤6 h, NE with Thompsons encephalopathy score >5	NA	17	16	WBC (phase changing material)	rectal	33.5	36.4
26	THIN study	Aker 2019 [31]	India	GA≥36 wk, BW>1800 g, age <5 h, perinatal asphyxia (umbilical cord or 1st h pH<7.0 or BD≥12), 5 min AS≤5, or need of PPV≥10 min at birth	Major congenital anomalies or imminent death anticipated	25	25	WBC (phase changing material)	rectal	33.5 ± 0.5	37.0 ± 0.5
27	TOBY trial	Azzopardi 2009 [30], Azzopardi 2014 [33], Campbell 2018 [37], Perrone 2010 [48], Roka 2011 [51], Rutherford 2010 [52]	UK	GA≥36 wk, at 10 min after birth, either AS≤5 or continued need for resuscitation or, within 60 min after birth, acidosis (umbilical-cord, arterial, or capillary pH<7.00 or BD≥16mmol/l); moderate-to-severe encephalopathy (lethargy, stupor, or coma) and either hypotonia, abnormal reflexes, absent or weak suck, or clinical seizures; abnormal background activity for ≥30 min or seizures (on aEEG)	Age >6 h, major congenital abnormalities that required surgery or were suggestive of chromosomal anomaly or syndromes that involve brain dysgenesis	163	162	WBC (cooling blanket)	rectal	33.0-34.0	37.0 ± 0.2
28	Yang	Yang 2020 [61]	China	Age <6 h; GA 37 wk and BW 2500 g; 1 min AS 3 and 5 min AS 5	Convulsions caused by electrolyte disorder, intracranial hemorrhage, brain injury caused by intrauterine infection, genetic and metabolic diseases, other congenital diseases; congenital malformation or congenital metabolic abnormality; suspicion of prenatal and intrapartum infection	62	30	SHC (cooling caps)	NA	28.0-30.0 (head skin temp); 34.5 ± 0.5 (body surface skin); 35.5 ± 0.5 (anal temp)	NA
29	Zhou	Zhou 2010 [62]	China	Age <6 h, GA 37 wk, BW 2500 g, with clinical evidence of perinatal hypoxia-ischemia or diagnosis of encephalopathy. AS≤3 at 1 min and ≤5 at 5 min; cord blood pH<7.0 or BD≤16 mmol/l; and need for resuscitation or ventilation at 5 min of age	Major congenital abnormalities; infection; other encephalopathy, severe anemia	138	118	SHC (cooling caps)	NP; rectal	34.0 ± 0.2 (NP); 34.5-35.0 (rectal)	36.0-37.5 (rectal)

EAC – external auditory canal, ECMO – extracorporeal membrane oxygenation, HELIX – hypothermia for moderate or severe neonatal encephalopathy in low-income and middle-income countries, HIE – hypoxic ischemic encephalopathy, HT – hypothermia, ICE – The Infant Cooling Evaluation, NA – not reported, NE – neonatal encephalopathy, NEST – neonatal ECMO Study of Temperature, NICHD – National Institute of Child Health and Human Development, NP – nasopharyngeal, SHC – selective head cooling, WBC – Whole body cooling, THIN – Therapeutic hypothermia for neonatal hypoxic-ischemic encephalopathy in India, TOBY – Total Body Hypothermia for Neonatal Encephalopathy Trial, wk – weeks

*In this trial, TH group had 4 subgroups with cooling to 36.5-36°C (n = 6): 35.9-35.5°C (n = 6): 35 ± 0.5°C (n = 6): 34.5 ± 0.5°C (n = 7). Only those with cooling to 34.5 ± 0.5°C were eligible for inclusion in this systematic review. The median time of initiation of intervention was within 4 hours of birth in the TH group; and 4.5 hours after birth in the control group.

†This study is the same as the Battin 2001 trial, however in this study data for TH group included participants cooled to temperature 35.0 ± 0.5°C (n = 6); or 34.5 ± 0.5°C (n = 7). Although, the latter conformed to the inclusion criteria of this review, outcome data could not be extracted separately for this group. Therefore, data from this study was unusable for meta-analysis.

Two of the 29 RCTs were multi-country trials [10,26]. Nine trials were conducted in India [28,31,35,36,38,44,45,49,60], six in China [39,46,47,59,61,62], four in the USA [26,29,41,53], two in the UK [30,42], and one each in Australia [43], Egypt [57], Germany [58], New Zealand [34], Turkey [32], and Uganda [50]. The sample sizes in the 29 trials ranged from 19 [53] to 408 [10]; with median (IQR) 93 (40, 158). Only 14 trials [10,26,27,29,30, 35,38,42,44,45,49,58,60,62] enrolled >100 participants each.

Nine RCTs [10,26,30,35,43,45,46,57,58] enrolled infants with moderate or severe encephalopathy, whereas another nine trials [27,29,41,44,47,49,59,60,62] also included some infants with mild encephalopathy. The proportion of such infants ranged from 0.2% to 23.3%. Eleven trials [28,31,32,34,36,38,39,42,50,53,61] did not describe the severity of encephalopathy. One study [48] presented data from a sub-group of participants reported in another study [30], hence data were extracted from the main publication.

### Mortality

**Figure 2:**
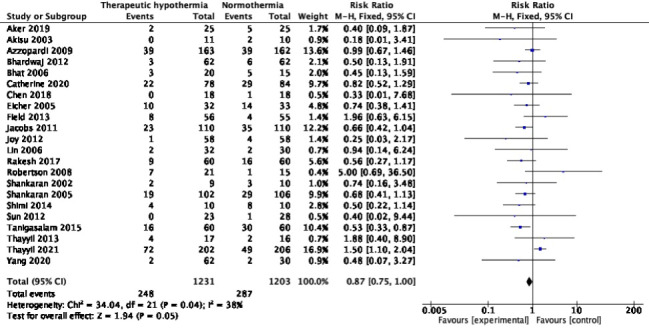
Meta-analysis of data on neonatal mortality (during the initial hospitalization).

Twenty-two studies with 2434 participants reported neonatal mortality during the initial hospitalization. The pooled RR was 0.87, (95% CI = 0.75, 1.00), *I^2^* = 38% (**Figure 2**). The absolute risk difference was -0.03 (95% CI = -0.06, 0.00), *I^2^* = 47%. One trial [58] reported mortality only during the intervention period, but not the entire hospitalization, hence its data was not pooled. Among the 22 trials, 21 showed an uncertain effect; only the HELIX trial [10] showed increased mortality. Excluding its data yielded a pooled RR of 0.74 (95% CI = 0.62, 0.87), *I^2^* = 0%.

**Figure 3:**
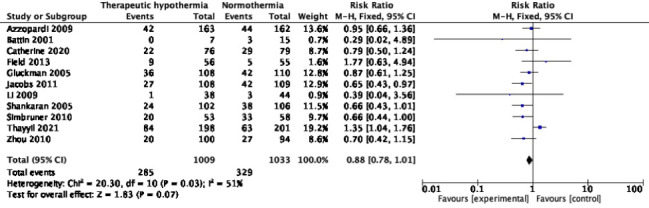
Meta-analysis of data on mortality at the age of 18-24 months.

Eleven trials with 2042 participants reported mortality at 18-24 months [10,26,27,29,30,34,38,42,46,58,62]; pooled RR (95% CI) was 0.88 (95% CI = 0.78, 1.01), *I^2^* = 51% (**Figure 3**). The absolute risk difference was -0.04 (95% CI = -0.08, 0.00), *I^2^* = 74%. Only one trial [27] showed statistically significant reduction, with a RR of 0.65 (95% CI = 0.43, 0.97); nine [26,29,30,34,38,42,46,58,62] showed statistically insignificant differences, and the HELIX trial [10] reported increased mortality, with an RR of 1.35 (95% CI = 1.04, 1.76). Excluding HELIX trial data [10] yielded a pooled RR of 0.77 (95% CI = 0.66, 0.90), *I^2^* = 0%. Two trials [58,62] had data missing for >10% enrolled participants. In the Simbruner 2010 [58] trial, 17.2% and 10.8% in the intervention and comparison arms had missing data. In the Zhou 2010 [62] trial, the respective proportions were 27.5% and 20.3%. Exclusion of these two trials did not remarkably change the pooled effect; RR was 0.93 (95% CI = 0.81, 1.07), *I^2^* = 53%.

**Figure 4:**
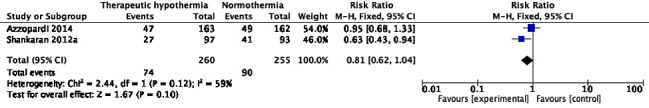
Meta-analysis of data on mortality between 5-10 years of age.

Only two studies [33,56] with 515 survivors reported mortality during childhood. Although one trial [56] reported statistically significant reduction, pooled RR (95% CI) was 0.81 (95% CI = 0.62, 1.04), *I^2^* = 59% (**Figure 4**). Random-effects model yielded RR 0.79 (95% CI = 0.53, 1.18). The absolute risk difference was -0.07 (95% CI = -0.15, 0.01), *I^2^* = 67%. No trial reported data on children older than ten years.

### Unfavourable neurological and/or neurodevelopmental outcomes (ie, disability)

**Figure 5:**
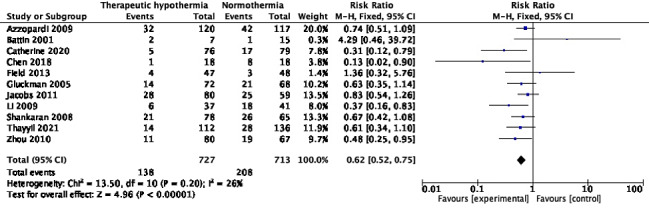
Meta-analysis of data on participants with neurologic disability at the age of 18-24 months.

Eleven trials [10,27,29,30,34,38,39,42,46,54,62] with 1440 participants reported this outcome at 18-24 months of age. Among these, nine trials used the Bayley Scales of Infant Development (second or third edition) [10,27,29,30,34,39,42,46,54], and one each used the Gesell Child Development Age Scale and the Gross Motor Function Classification System (GMFCS) [62], and Developmental Assessment Scale for Indian Infants [38]. Although only three trials [38,39,62] showed statistically significant reduction, whereas the other eight were inconclusive, pooled RR (95% CI) was 0.62 (95% CI = 0.52, 0.75), *I^2^* = 26% (**Figure 5**). The absolute risk difference was -0.11 (95% CI = -0.15, -0.07), *I^2^* = 46%. Four trials [27,29,46,54] had missing data in >10% survivors in at least one of the trial arms. Additionally, two trials had >10% difference in inter-group attrition. In the Jacobs 2011 trial [27], data were missing in 3.6% and 14.5% of survivors in the intervention and comparison groups. The respective proportions in the Li 2009 trial [46] were 17.8% and 6.8%.

**Figure 6:**
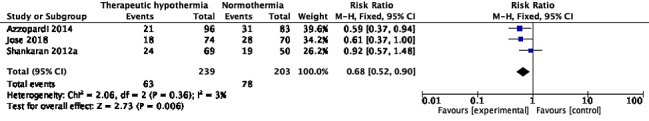
Meta-analysis of data on participants with neurologic disability between 5-10 years of age.

Three publications [33,44,56] presented the proportion with neurological disability during childhood, among 442 survivors; pooled RR was 0.68 (95% CI = 0.52, 0.90), *I^2^* = 3% (**Figure 6**). The absolute risk difference was -0.12 (95% CI = -0.21, -0.04), *I^2^* = 0%. The denominators in two of these [33,56] were less than the number of survivors, suggesting missing data. In the third publication [44], the originally randomized number was unavailable. There were no studies reporting the outcome at 10 years of age.

### Mortality or disability

**Figure 7:**
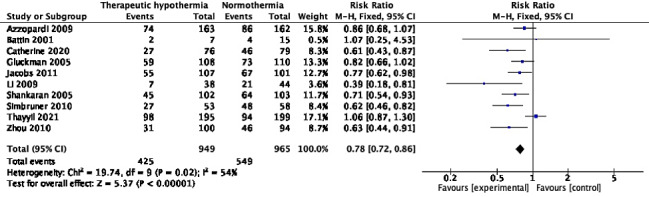
Meta-analysis of data on participants with death or neurologic disability at the age of 18-24 months.

Ten trials with 1914 participants reported the composite outcome of death or disability at 18-24 months of age [10,26,27,29,30,34,38,46,58,62]. Pooled RR (95% CI) was 0.78 (95% CI = 0.72, 0.86), *I^2^* = 54% (**Figure 7**). Random-effects model yielded a RR of 0.75 (95% CI = 0.66, 0.87). The absolute risk difference was -0.12 (95% CI = -0.17, -0.08), *I^2^* = 59%. Unlike when the two outcomes were analysed separately, TH showed statistically significant improvement in the composite outcome in six of ten trials [26,27,38,46,58,62], and none including the HELIX trial [10] showed increased risk. Three trials [47,58,62] had data missing in >10% of participants in at least one arm. Excluding these trials yielded RR 0.84 (95% CI = 0.76, 0.92), *I^2^* = 43%. In addition, the difference in attrition between the trial arms was >10% in the Li 2009 trial [46]. In the Zhou 2010 trial [62], the proportions with missing data were 27.5% in the intervention arm and 20.3% in the comparison arm. Exclusion of these trials did not significant alter the pooled effect; RR was 0.60 (95% CI = 0.46, 0.78), *I^2^* = 41%.

### Cerebral palsy

**Figure 8:**
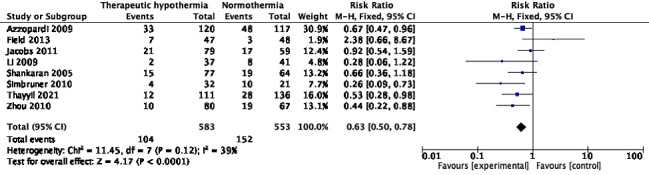
Meta-analysis of data on participants with cerebral palsy at the age of 18-24 months.

Eight trials (1136 participants) reported the proportion of infants with cerebral palsy (CP) at 18-24 months of age [10,26,27,30,42,46,58,62]. Although only four [10,30,58,62] independently showed statistically significant reduction, the pooled RR was 0.63 (95% CI = 0.50, 0.78), *I^2^* = 39% (**Figure 8**). Two trials [46,62] had data missing from >10% survivors in at least one arm, but their exclusion did not change the pooled effect; the RR was 0.68 (95% CI = 0.54, 0.86), *I^2^* = 43%. The absolute risk difference across the 8 trials was -0.10 (95% CI = -0.15, -0.06), *I^2^* = 55%.

**Figure 9:**
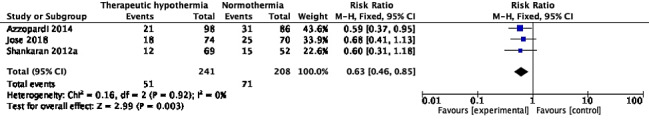
Meta-analysis of data on participants with cerebral palsy between 5-10 years of age.

Three studies [33,44,56] (449 survivors) reported the proportions with cerebral palsy during childhood; pooled RR was 0.63 (95% CI = 0.46, 0.85), *I^2^* = 0% (**Figure 9**). The denominators in two [33,56] of these were less than the number of survivors, suggesting missing data. In the third publication [44], the number originally randomized was unavailable. The absolute risk difference across the 3 studies was -0.13 (95% CI = -0.21, -0.04), *I^2^* = 0%. No studies reported cerebral palsy at 10 years of age.

### Other outcomes

#### Seizures

**Figure 10:**
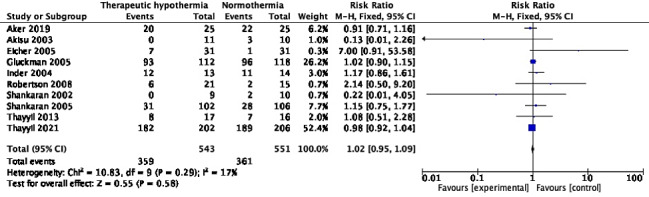
Meta-analysis of data on participants with neonatal seizures (during the initial hospitalization).

Ten trials [10,26,28,29,31,32,41,43,50,53] with 1094 participants reported neonatal seizures. The pooled RR was 1.02 (95% CI = 0.95, 1.09), *I^2^* = 17% (**Figure 10**). The absolute risk difference was 0.01 (95% CI = -0.03, 0.06), *I^2^* = 42%

**Figure 11:**
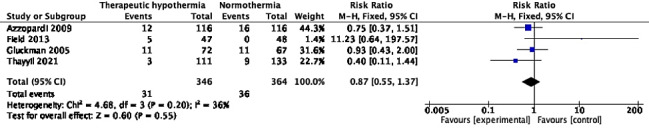
Meta-analysis of data on participants with seizures at the age of 18-24 months (ie, infantile epilepsy).

Only four trials (710 participants) reported the proportion of infants with seizures at 18-24 months, ie, infantile epilepsy as a sequel to neonatal encephalopathy [10,29,30,42]. The pooled RR was 0.87 (95% CI = 0.55, 1.37), *I^2^* = 36% (**Figure 11**). The absolute risk difference was -0.01 (95% CI = -0.06, 0.03), *I^2^* = 60%. One trial [29] had data missing from >10% survivors, however its exclusion did not change the pooled effect: 0.84 (95% CI = 0.48, 1.48), *I^2^* = 56%.

Only one [56] publication with 117 children presented data on seizures during childhood (ie, childhood epilepsy); there was no statistically significant impact, and RR was 0.65 (95% CI = 0.25, 1.68). The absolute risk difference was -0.06 (95% CI = -0.18, 0.07), N = 1, n = 117.

#### Length of hospital stay

Nine trials reported length of hospital stay during the initial hospitalization; five [26,32,35,53,58] yielded a pooled mean difference (95% CI) of -0.82 days (95% CI = -1.65, 0.02). The other four presented data as median (IQR) [10,30,38,42]. Although their hospitalization durations varied widely, they were comparable in both arms.

#### EEG abnormalities

**Figure 12:**
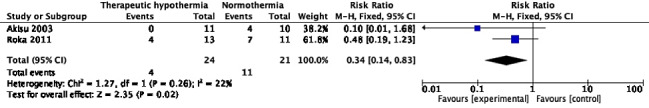
Meta-analysis of data on participants with EEG abnormalities during the neonatal period.

Only two publications [32,51] with 45 participants reported the proportion with EEG abnormalities during the initial hospitalization. One trial [32] performed EEG, 4-10 days after birth, whereas the other performed aEEG during the first 72 hours and calculated the proportion with persisting abnormalities. Pooled RR (95% CI) was 0.34 (95% CI = 0.14, 0.83), *I^2^* = 22% (**Figure 12**). The absolute risk difference was -0.36 (95% CI = -0.62, -0.10), *I^2^* = 0%.

#### Abnormalities on MRI

**Figure 13:**
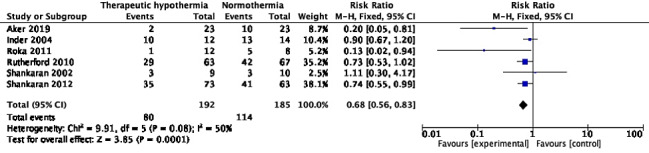
Meta-analysis of data on participants with ‘any MRI lesions’ during the neonatal period.

**Figure 14:**
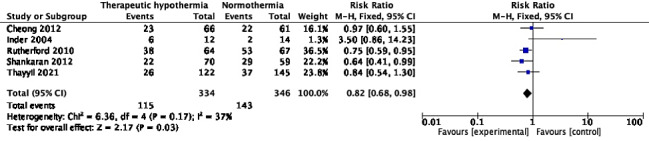
Meta-analysis of data on participants with basal ganglia lesions or thalamic injury on MRI, during the neonatal period.

**Figure 15:**
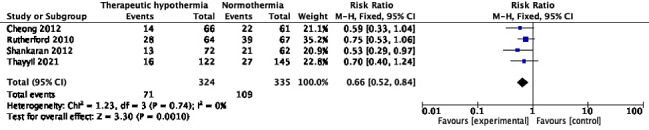
Meta-analysis of data on participants with PLIC lesions on MRI during the neonatal period.

**Figure 16:**
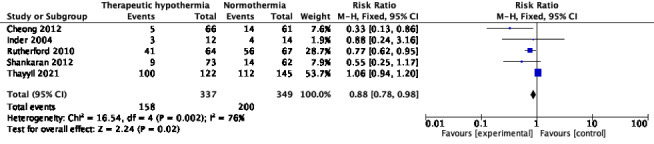
Meta-analysis of data on participants with white matter injury on MRI during the neonatal period.

Eight trials reported MRI abnormalities during the initial hospitalization [10,31,40,43,51-53,55]. The timing of MRI varied as follows: during 7-14 days after birth [10], on the 5^th^ day after birth [31], within the first 10 days of birth [40], during the first 7 days of life [43], between days 5-14 of life [51], within the first 4 weeks of birth [52], and by 44 weeks of post-menstrual age [53,55]. The pooled RR for number of infants with “any MRI abnormality” was 0.68 (95% CI = 0.56, 0.83), *I^2^* = 50%, 6 trials, 377 participants (**Figure 13**). Random-effects model yield RR of 0.73 (95% CI = 0.54, 0.98). The absolute risk difference was -0.19 (95% CI = -0.29, -0.10), *I^2^* = 28%. Three trials [31,51,55] showed a lower proportion, whereas the others [43,52,53] reported uncertain effect. MRI abnormalities in the basal ganglia region, or thalamic injury were reported in five trials [10,40,43,52,55] (680 participants); pooled RR was 0.82 (95% CI = 0.68, 0.98), I^2^ = 37% (**Figure 14**). The absolute risk difference was -0.08 (95% CI = -0.14, -0.01), *I^2^* = 64%. Two of these trials [52,55] showed statistically significant reduction. Four trials [10,40,52,55] with 659 participants reported those with lesions in the posterior limb of the internal capsule (PLIC). Although only one [55] showed statistically significant reduction with TH, pooled RR was 0.66 (95% CI = 0.52, 0.84), *I^2^* = 0% (**Figure 15**). The absolute risk difference was -0.11 (95% CI = -0.18, -0.05), *I^2^* = 0%. White matter injury was reported in various ways in five trials [10,40,43,52,55] (686 participants). Although a statistically significant reduction was seen in only two trials [40,52], pooled RR was 0.88 (95% CI = 0.78, 0.98), *I^2^* = 76% (**Figure 16**). Random-effects model yielded RR 0.76 (95% CI = 0.54, 1.09). The absolute risk difference was -0.07 (95% CI = -0.13, -0.01), *I^2^* = 62%.

#### Quality of life

A single trial [37] presented information on quality of life during childhood using various scoring systems. The proportion with Health Utilities Index (HUI3) score was not different in the two arms, RR was 0.76 (95% CI = 0.55, 1.04); and the mean difference of scores was also similar; 0.09 (95% CI = -0.06, 0.23).

### Subgroup analysis

We examined the outcomes by study setting (**Table 2**). Neonatal mortality and neonatal seizures did not show statistically significant inter-group differences, in any of the four types of countries/settings. TH significantly reduced mortality at 18-24 months in HIC but did not show statistically significant differences in UMIC or LMIC. Similarly, the composite outcome of death or disability at 18-24 months was significantly lowered in HIC and UMIC, but not LMIC. However, neurological disability and cerebral palsy at 18-24 months showed statistically significant reduction across settings.

**Table 2:** Analysis of outcomes by country/setting of the trials*

Outcome	Overall	HIC	UMIC	LMIC	LIC
**Neonatal mortality**	0.87 (0.75, 1.00), N = 22, n = 2434, *I^2^* = 38%	0.82 (0.65, 1.03), N = 6, n = 948, *I^2^*= 0%.	0.47 (0.16, 1.32), N = 5, n = 262, *I^2^* = 0%	0.89 (0.74, 1.09), N = 10, n = 1188, *I^2^* = 62%	5.00 (0.69, 36.50), N = 1, n = 36
**Mortality at 18-24 mo**	0.88 (0.78, 1.01), N = 11, n = 2042, *I^2^* = 51%	0.79 (0.66, 0.93), N = 7, n = 1212, *I^2^* = 7%	0.67 (0.41, 1.09), N = 2, n = 276, *I^2^* = 0%	1.18 (0.94, 1.47), N = 2, n = 554, *I^2^* = 75%	No trial
**Mortality at 5-10 y of age**	0.81 (0.62, 1.04), N = 2, n = 515, *I^2^* = 59%	0.81 (0.62, 1.04), N = 2, n = 515, *I^2^* = 59%	No trial	No trial	No trial
**Neurological disability at 18-24 mo**	0.62 (0.52, 0.75), N = 11, n = 1440, *I^2^* = 26%	0.76 (0.61, 0.95), N = 6, n = 782, *I^2^* = 0%	0.38 (0.23, 0.62), N = 3, n = 261, *I^2^* = 0%	0.49 (0.30, 0.80), N = 2, n = 403, *I^2^* = 32%	No trial
**Neurological disability at 5-10 y of age**	0.68 (0.52, 0.90), N = 3, n = 442, *I^2^* = 3%.	0.72 (0.51, 1.00), N = 2, n = 298, *I^2^* = 42%	No trial	0.61 (0.37, 1.00), N = 1, n = 144	No trial
**Mortality or disability at 18-24 mo**	0.78 (0.72, 0.86), N = 10, n = 1914, *I^2^* = 54%	0.77 (0.69, 0.86), N = 6, n = 1089, *I^2^* = 0%	0.56 (0.41, 0.78), N = 2, n = 276, *I^2^* = 30%	0.92 (0.77, 1.09), N = 2, n = 549, I^2^ = 86%	No trial
**Cerebral palsy at 18-24 mo**	0.63 (0.50, 0.78), N = 8, n = 1136, *I^2^* = 39%	0.72 (0.56, 0.92), N = 5, n = 664, *I^2^* = 51%	0.40 (0.21, 0.75), N = 2, n = 225, *I^2^* = 0%	0.53 (0.28, 0.98), N = 1, n = 247	No trial
**Cerebral palsy at 5-10 y of age**	0.63 (0.46, 0.85), N = 3, n = 449, *I^2^* = 0%	0.60 (0.41, 0.88), N = 2, n = 305, *I^2^* = 0%	No trial	0.68 (0.41, 1.13), N = 1, n = 144	No trial
**Neonatal seizures**	1.02 (0.95, 1.09), N = 10, n = 1094, *I^2^* = 17%	1.09 (0.95, 1.24), N = 5, n = 546, I^2^ = 31%	0.13 (0.01, 2.26), N = 1, n = 21	0.98 (0.92, 1.04), N = 3, n = 491, *I^2^* = 0%	2.14 (0.50, 9.20), N = 1, n = 36
**Seizures at 18-24 mo (infantile epilepsy)**	0.87 (0.55, 1.37), N = 4, n = 710, *I^2^* = 36%	1.01 (0.62, 1.65), N = 3, n = 466, *I^2^* = 42%	No trial	0.40 (0.11, 1.44), N = 1, n = 244	No trial
**Seizures at 5-10 y of age (childhood epilepsy)**	0.65 (0.25, 1.68), N = 1, n = 117	0.65 (0.25, 1.68), N = 1, n = 117	No trial	No trial	No trial

HIC – high-income countries, UMIC – upper middle-income countries, LMIC – lower middle-income countries, LIC – low-income countries, mo – months, y – years

*All data are presented as risk ratios (RR) with 95% confidence interval. ‘N’ represents the number of trials, and ‘n’ represents the number of participants.

Subgroup analysis by type of cooling (**Table 3**) showed statistically insignificant inter-group differences between WBC and SHC, for mortality (neonatal and at 18-24 months) and seizures at any age. Other outcomes at 18-24 months, namely neurological disability, composite of mortality or disability, and cerebral palsy, were all improved with TH, irrespective of whether the whole body or only the head was cooled.

**Table 3:** Analysis of outcomes by type of cooling*

Outcome	Overall	Whole-body cooling	Selective head cooling
**Neonatal mortality**	0.87 (0.75, 1.00), N = 22, n = 2434, *I^2^* = 38%	0.88 (0.76, 1.02), N = 17, n = 2172, I^2^ = 50%	0.47 (0.16, 1.32), N = 5, n = 262, *I^2^ *= 0%
**Mortality at 18-24 mo**	0.88 (0.78, 1.01), N = 11, n = 2042, *I^2^* = 51%	0.91 (0.79, 1.06), N = 8, n = 1608, *I^2^* = 62%	0.79 (0.59, 1.05), N = 3, n = 434, *I^2^* = 0%
**Mortality at 5-10 y of age**	0.81 (0.62, 1.04), N = 2, n = 515, I^2^ = 59%	0.81 (0.62, 1.04), N = 2, n = 515, I^2^ = 59%	No trial
**Neurological disability at 18-24 mo**	0.62 (0.52, 0.75), N = 11, n = 1440, *I^2^* = 26%	0.65 (0.53, 0.80), N = 7, n = 1095, *I^2^* = 15%	0.54 (0.36, 0.81), N = 4, n = 345, *I^2^* = 48%
**Neurological disability at 5-10 y of age**	0.68 (0.52, 0.90), N = 3, n = 442, *I^2^* = 3%	0.68 (0.52, 0.90), N = 3, n = 442, *I^2^* = 3%	No trial
**Mortality or disability at 18-24 mo**	0.78 (0.72, 0.86), N = 10, n = 1914, *I^2^* = 54%	0.79 (0.72, 0.88), N = 7, n = 1480, *I^2^* = 67%	0.75 (0.63, 0.91), N = 3, n = 434, *I^2^* = 0%
**Cerebral palsy at 18-24 mo**	0.63 (0.50, 0.78), N = 8, n = 1136, *I^2^* = 39%	0.66 (0.52, 0.83), N = 7, n = 989, *I^2^* = 41%	0.44 (0.22, 0.88), N = 1, n = 147
**Cerebral palsy at 5-10 y of age**	0.63 (0.46, 0.85), N = 3, n = 449, *I^2^* = 0%	0.63 (0.46, 0.85), N = 3, n = 449, *I^2^* = 0%	No trial
**Neonatal seizures**	1.02 (0.95, 1.09), N = 10, n = 1094, *I^2^* = 17%	1.03 (0.95, 1.11), N = 8, n = 843, *I^2^* = 23%	0.99 (0.87, 1.12), N = 2, n = 251, *I^2^* = 55%
**Seizures at 18-24 mo (infantile epilepsy)**	0.87 (0.55, 1.37), N = 4, n = 710, *I^2^* = 36%	0.84 (0.48, 1.48), N = 3, n = 571, *I^2^* = 56%	0.93 (0.43, 2.00), N = 1, n = 139
**Seizures at 5-10 y of age (childhood epilepsy)**	0.65 (0.25, 1.68), N = 1, n = 117	0.65 (0.25, 1.68), N = 1, n = 117	No trial

mo – months, y – years

*All data are presented as risk ratios (RR) with 95% CI. ‘N’ represents the number of trials, and ‘n’ represents the number of participants.

### Sensitivity analysis

Sensitivity analysis excluding trials with moderate/high RoB (from the analysis) did not change the overall result for major clinical outcomes, although the magnitude of effect diminished for some outcomes (**Table 4**). However, the exclusion changed three statistically significant differences in MRI outcomes to statistically insignificant differences (**Table 4**). Examination of pooled risk ratios among trials with low RoB against those with moderate or high RoB showed that TH reduced neonatal mortality and mortality at 18-24 months in trials with moderate/high RoB, but not in trials with low RoB (**Table 4**). However neurological disability, cerebral palsy, and the composite outcome of disability or mortality at 18-24 months showed benefit with TH in both types of trials, although the magnitude was less in low RoB trials.

**Table 4:** Analysis of outcomes by risk of bias within the trials*

Outcome	Overall	Trials with low risk of bias	Trials with moderate or high risk of bias
**Neonatal mortality**	0.87 (0.75, 1.00), N = 22, n = 2434, *I^2^ *= 38%	0.97 (0.80, 1.16), N = 7, n = 1475, *I^2^* = 62%	0.71 (0.55, 0.91), N = 15, n = 959, *I^2^* = 0%
**Mortality at 18-24 mo**	0.88 (0.78, 1.01), N = 11, n = 2042, *I^2^* = 51%	0.96 (0.83, 1.13), N = 6, n = 1336, *I^2^* = 58%	0.72 (0.56, 0.92), N = 5, n = 706, *I^2^* = 0%
**Mortality at 5-10 y of age**	0.81 (0.62, 1.04), N = 2, n = 515, *I^2^* = 59%	No trial	0.81 (0.62, 1.04), N = 2, n = 515, *I^2^* = 59%
**Neurological disability at 18-24 mo**	0.62 (0.52, 0.75), N = 11, n = 1440, *I^2^* = 26%	0.68 (0.54, 0.85), N = 6, n = 941, I^2^ = 24%	0.52 (0.38, 0.73), N = 5, n = 499, *I^2^* = 28%
**Neurological disability at 5-10 y of age**	0.68 (0.52, 0.90), N = 3, n = 442, *I^2^* = 3%	No trial	0.68 (0.52, 0.90), N = 3, n = 442, *I^2^* = 3%
**Mortality or disability at 18-24 mo**	0.78 (0.72, 0.86), N = 10, n = 1914, *I^2^* = 54%	0.86 (0.77, 0.95), N = 6, n = 1322, *I^2^* = 43%	0.63 (0.53, 0.75), N = 4, n = 592, *I^2^* = 0%
**Cerebral palsy at 18-24 mo**	0.63 (0.50, 0.78), N = 8, n = 1136, *I^2^* = 39%.	0.68 (0.52, 0.90), N = 3, n = 622, I^2^ = 0%	0.55 (0.38, 0.79), N = 5, n = 514, *I^2^* = 53%
**Cerebral palsy at 5-10 y of age**	0.63 (0.46, 0.85), N = 3, n = 449, *I^2^* = 0%	No trial	0.63 (0.46, 0.85), N = 3, n = 449, *I^2^*= 0%
**Neonatal seizures**	1.02 (0.95, 1.09), N = 10, n = 1094, *I^2^* = 17%	0.99 (0.94, 1.05), N = 2, n = 638, *I^2^* = 0%	1.11 (0.89, 1.38), N = 8, n = 456, *I^2^* = 30%
**Seizures at 18-24 mo (infantile epilepsy)**	0.87 (0.55, 1.37), N = 4, n = 710, *I^2^* = 36%	0.73 (0.45, 1.17). N = 3, n = 615, *I^2^* = 0%	11.23 (0.64, 197.57), N = 1, n = 95
**Seizures at 5-10 y of age (childhood epilepsy)**	0.65 (0.25, 1.68), N = 1, n = 117	No trial	0.65 (0.25, 1.68), N = 1, n = 117

mo – months, y – years

*All data are presented as risk ratios (RR) with 95% CI. ‘N’ represents the number of trials, and ‘n’ represents the number of participants.

## DISCUSSION

This up-to-date systematic review showed that therapeutic hypothermia implemented for neonatal encephalopathy, did not result in statistically significant reductions in mortality during the neonatal period, infancy or later childhood. However, it reduced neurologic disability and cerebral palsy in infancy and childhood, resulting in reduction in the composite outcome of mortality or disability, despite absence of conclusive benefit on mortality alone. EEG abnormalities and multiple MRI outcomes were better in neonates who received TH. However, there was no statistically significant impact on seizures during the neonatal period, infantile epilepsy, or childhood epilepsy.

While the type of cooling (ie, WBC or SHC) did not affect the results, the setting where TH was implemented was relevant. TH reduced mortality at 18-24 months in high income countries, but not in other settings. While neonatal mortality and seizures were not reduced in any setting, disability and cerebral palsy in infancy were reduced in all settings.

More important, reduction in mortality reported in previous systematic reviews [1,2] was influenced by trials with higher risk of bias.

Thus, this systematic review uncovered several novel findings that contradict previous reviews [1,2]. This is partly because of the availability of new trials, notably the HELIX trial [10], but also due to methodological errors in the previous reviews. The Cochrane review combined immediate and later mortality in the same meta-analysis [1]. The later review failed to include some eligible trials, duplicated data from some trials, presented data from non-existent trials, combined immediate and later mortality, and even expressed relative risk with negative integers [2].

The HELIX trial [10] reported increased mortality (neonatal and infancy) with TH, in stark contrast to previous trials. This RCT was one of the best conducted trials with multiple methodological refinements, strict definitions, largest sample size, extremely low attrition rate, and low risk of bias. Extensive critical appraisal did not identify any major limitations [18], although some concerns were raised about the inclusion of out-born infants, slightly delayed initiation of cooling (though within the accepted limit of 6 hours), and possibly diverse causes of hypoxic encephalopathy in low-resource settings [19].

This systematic review had several strengths notably exhaustive literature search across published and grey literature, inclusion of the largest cohort of trials to date, searching and data extraction in duplicate, careful extraction of data meeting the review criteria (rather than including data reported by trials), and undertaking multiple subgroup and sensitivity analyses. There were no deviations from the protocol [21]. In fact, several additional outcomes were also presented. This fosters high confidence in the review findings.

We acknowledge several limitations in our review. We could not search Chinese language databases, or conference proceedings. We could not obtain individual participant data, or missing data for intention-to-treat analyses. In the protocol, we mentioned that randomized controlled trials would be included, but did not specify how quasi or pseudo randomized studies, would be handled. Analysis of the randomization method identified that 18 trials used an appropriate method of randomization, 1 trial used a quasi-randomization method, and 10 trials had an unclear method. Thus, the included trials had some quasi/pseudo randomized studies. The impact of this is evident from the differences in some outcomes among trials with low vs higher RoB.

The effect of therapeutic hypothermia may also be influenced by several factors such as the proportion of outborn neonates in studies, proportion with severe encephalopathy, method of cooling (servo vs non-servo), and severity of asphyxia. For example, 4 trials excluded outborn neonates, 14 trials included them (but only 8 of them reported the proportion of outborn babies), and 11 trials did not provide any information, Similarly, 15 studies reported data of participants with only severe or moderate neonatal encephalopathy, 9 studies included those with mild encephalopathy also, but the proportion was <25% of the total, and 15 studies did not report details of severity. Among these 15, data on Apgar score and/or cord blood parameters suggested severe disease in some (Table S2 in the **Online Supplementary Document**). Thirteen studies did not report any data on Apgar scores or cord blood parameters, whereas 26 studies reported either or both (Table S2 in the **Online Supplementary Document**). In the absence of individual patient data, it is not possible to account for these factors.

Before initiating this review, we listed the primary outcome as mortality or neurologic disability at the age of 18-24 months, in alignment with previous systematic reviews [1,2], and major trials [1,2,10,26,29,30]. Although the composite outcome provides useful information, we believe that it is skewed by the beneficial effects of TH on neurologic outcomes, masking the lack of statistically significant impact on mortality.

Adverse effects of therapeutic hypothermia were reported in various ways, and at various time points, in several trials. Although these are very important to consider, for making informed decisions (at the practice as well as policy levels), in this systematic review, we focused on evidence of efficacy, and did not examine adverse events.

We expected attrition in trials would bias the results in favor of the intervention, but did not observe this for most outcomes.

Finally, is more research required on therapeutic hypothermia for neonatal encephalopathy? Some experts would argue that more trials should be conducted until an optimal information size is achieved following which, further research can be discontinued. This would be very expensive in terms of time and resources. Instead, we suggest that research in local health care systems in resource-constrained settings, could focus on resolving issues such as which neonates are most likely to benefit from TH, predictors of failure, and of course primary prevention.

## CONCLUSIONS

This up-to-date systematic review of randomized controlled trials confirmed that therapeutic hypothermia implemented for neonatal encephalopathy reduces neurologic disability and cerebral palsy in diverse settings. However, it has an unclear effect on neonatal, infantile, and childhood mortality. It also does not impact neonatal seizures, or epilepsy during infancy and childhood. The previously reported reduction in mortality was associated with trials of lower methodological quality, but not substantiated by trials with high(er) quality.

## Additional material


Online Supplementary Document
