# Supplementary material for: Therapeutic hypothermia in neonatal hypoxic encephalopathy: A systematic review and meta-analysis
Source: J Glob Health. 2022 Apr 9;12:04030. doi: 10.7189/jogh.12.04030 (PMC8994481; doi:10.7189/jogh.12.04030)
Supplement: Online Supplementary Document [file jogh-12-04030-s001.pdf]

Supplementary Table 1. Detailed search strategy in representative databases

Database	URL Link	Search Strategy
Medline (through Pubmed)	https://pubmed.ncbi.nlm.nih.gov/advanced/	(Neonate (MeSH Terms) OR newborn OR perinatal OR infant) AND (hypothermia (MeSH Terms) OR (cool OR cooling OR temperature OR body temperature) AND (death (MeSH Terms) OR mortality) AND (asphyxia (MeSH Terms) OR hypoxic-ischemic OR hypoxic-ischaemic OR hypoxia OR brain OR encephalopathy AND thrapy **Filters:** Randomized Controlled Trial
Cochrane Library (CENTRAL)	https://neonatal.cochrane.org/resources-authors/author-resources-new-reviews	1. MESH DESCRIPTOR Infant, Newborn EXPLODE ALL AND CENTRAL:TARGET<br>2. infant or infants or infant's or "infants" or infantile or infancy or newborn* or "new born" or "new borns" or "newly born" or neonat* or<br>baby*<br>3. therapeutic hypothermia or cooling or neonatal cooling or whole body cooling or selective head cooling<br>(#1 or #2) AND #3
CINAHL	https://www.ebsco.com/products/research-databases/cinahl-complete	(infant or infants or infant's or infantile or infancy or newborn* or "new born" or "new borns" or "newly born" or neonat* or baby* or babies AND (therapeutic hypothermia or cooling or neonatal cooling or whole body cooling or selective head cooling) AND (randomized controlled trial OR controlled clinical trial OR randomized OR randomised OR placebo OR clinical trials<br>as topic OR randomly OR trial OR PT clinical trial)

Supplementary Table 2. Detailed description of the included studies

**No.**	**Publication**	**Severity of NE/HIE (Severe, moderate, mild)**		**Birth weight (BW) in g; Mean (SD)**		**Gestation Age (GA) in weeks; Mean (SD)**		**Apgar Scores (AS) at 5 min**		**First arterial blood gas (pH), Mean (SD)**		**First arterial blood gas (BD), Mean (SD)**	
		**TH**	**Control**	**TH**	**Control**	**TH**	**Control**	**TH**	**Control**	**TH**	**Control**	**TH**	**Control**
1	Aker 2019 [31]	NA	NA	2911 (483)	2960 (553)	39·1 (1·3)	39·2 (1·4)	NA	NA	6·81 (0·12)	6·93 (0·18)	-19·6 (3·2)*	-16·5 (4·6)
2	Akisu 2003 [32]	NA	NA	3270 (520)	3410 (575)	39·1 (0·9)	39·3 (1·4)	4·1 (1)	4·3 (1)	7·03 (0·1)	7·02 (0·1)	-15·3 (8) *	14·2 (10·2)
3	Azzopardi 2009 [30]	98,65,0	95,67,0	†3450 (2957-3873)	†3350 (3044-3729)	†40·3 (39·1-41·3)	†40·1 (38·8-41·1)	NA	NA	NA	NA	NA	NA
4	Azzopardi 2014 [33]‡	83,62,0	78,57,0	†3467 (3053-3883)	†3351 (3060-3700)	†40·3 (39·3-41·3)	†40·1 (39·0-41·1)	NA	NA	NA	NA	NA	NA
5	Battin 2001 [34]§	NA	NA	3892 (628)	3458 (438)	39·8 (0·8)	39·4 (1·7)	3 (0-6)	5 (2-7)	7·02 (0·13)	6·85 (0·25)	-14 (5·8)*	-19·8 (8·7)*

6	Battin 2003 [63] ¶	NA	NA	3333 (496)	3371 (383)	40 (2)	39·8 (1·4)	5 (2-7)	5 (2-7)	6·88 (0·19)	6·88 (0·2)	-19 (7·4)	-19 (8)
7	Bharadwaj 2012 [35]	7,55,0	8,54,0	2967 (380)	2899 (363)	39·8 (1·5)	40 (1·4)	NA	NA	7·09 (0·08)	7·08 (0·1)	-17·6 (3·8)*	-17·6 (3·9)*
8	Bhat 2006 [36]	NA	NA	NA	NA	NA	NA	NA	NA	NA	NA	NA	NA
9	Campbell 2018 [37]	NA	NA	†3460 (3172-3828)	†3400(3200-3850)	†40·1 (39·0-41·0)	†40·2 (39·3-41·4)	NA	NA	NA	NA	NA	NA
10	Catherine 2020 [38]	NA	NA	2875 (421)	2805 (399)	39·6 (1·6)	39·7 (1·7)	5 (4-6)	5 (5-6)	NA	NA	NA	NA
11	Chen 2018 [39] ‖	NA	NA	NA	NA	NA	NA	NA	NA	NA	NA	NA	NA
12	Cheong 2012 [40] **	14,39,0	14,31,0	3318 (635)	3489 (597)	38·9 (1·8)	38·9 (1·6)	NA	NA	6·9 (0·2)	6·9 (0·2)	-20·8 (7·9)*	-19·2 (9·6)
13	Eicher 2005 [41]	25,05,01	25,05,01	3241 (775)	3550 (819)	38·8 (1·9)	39·1 (1·4)	2	2	6·95 (0·19)	6·96 (0·23)	-18 (8·3)	-16 (7·5)
14	El Shimi 2014 [57]‖‖	4,6,0	4,6,0	NA	NA	NA	NA	NA	NA	NA	NA	NA	NA
15	Field 2013 [42] ††	NA	NA	3415 (601)	3480 (574)	39·6 (1·7)	40·3 (1·5)	7 (5-9)	8 (5-9)	NA	NA	NA	NA
16	Gluckman 2005 [29]	42,63,07	32,76,09	3399 (663)	3504 (625)	38·9 (1·6)	39·1 (1·4)	NA	NA	6·9 (0·2)	6·9 (0·2)	21·0 (32·0-3·6)	20·4 (35·2-3·9)
17	Inder 2004 [43]	5,8,0	5,9,0	NA	NA	NA	NA	NA	NA	NA	NA	NA	NA
18	Jacobs 2011 [26]	30,63,17	29,54,25	3348 (598)	3515 (611)	39·0 (1·8)	39·2 (1·7)	3 (1-4)	3 (1-4)	6·9 (0·2)	6·9 (0·2)	-20·4 (7·7) *	-19·0 (9·2_
19	Jose 2018[44]	22,51,1	4,39,3	3529 (598)	3278 (557)	NA	NA	NA	NA	6·7 (0·3)	6·6 (0·3)	17·5 (6·4)	21·5 (4·6)
20	Joy 2012 [45]	9,49,0	7,51,0	2840 (350)	2910 (360)	NA	NA	NA	NA	7·06 (0·05)	7·09 (0·12)	19·41 (5·81)	19·05 (5·07)
21	Li 2009 [46]‡‡	24,16,0	34,12,0	3342 (513)	3241 (436)	39·1 (1·0)	39·1 (1·6)	6·4 (1·5)	6·6 (1·4)	7·31 (0·11)	7·30 (0·15)	8·8 (5·1)	7·7 (3·8)
22	Lin 2006 [47]	7,16,7	6,15,7	3310 (470)	3430 (520)	38·7 (1·3)	39·1 (1·6)	3 (1)	3(1)	7·05 (0·12)	7·07 (0·11)	15·9 (4·6)	15·6 (4·8)
23	Perrone 2010 [48]	2,7,1	4,6,1	3550 (2300-4390)	3450 (2540-4040)	37·5 (36-41)	39·0 (36-41)	NA	NA	NA	NA	NA	NA

24	Rakesh 2017 [49]	18,41,1	14,44,2	2720 (450)	2800 (610)	38·7 (4·7)	39·1 (4·8)	NA	NA	6·94 (0·22)	6·92 (0·14)	16·12 (2·9) *	15·8 (3·2)
25	Robertson 2008 [50]	NA	NA	3300 (550)	3200 (268)	38 (1·45)	38 (1·38)	4·7	5·2	NA	NA	NA	NA
26	Roka 2011 [51]‡	NA	NA	NA	NA	NA	NA	NA	NA	NA	NA	NA	NA
27	Rutherford 2010 [52]‡	NA	NA	†3450 (2957-3873)	†3350 (3044-3729)	†40·3 (39·1-41·3)	†40·1 (38·8-41·1)	NA	NA	NA	NA	NA	NA
28	Shankaran 2002 [53]	NA	NA	3186 (431)	3269 (598)	38 (1)	40 (1)	NA	NA	6·89 (0·08)	6·86 (0·08)	18·5 (2·8)	18·8 (3·3)
29	Shankaran 2005 [27]§§	32,69,0	40,66,0	3385 (617)	3370 (645)	NA	NA	NA	NA	6·9 (0·2)	6·8 (0·2)	18·5 (6·7)	19·9 (8·7)
30	Shankaran 2008 [54]¶¶	32,69,0	40,66,0	3385 (617)	3370 (645)	NA	NA	NA	NA	6·9 (0·2)	6·8 (0·2)	18·5 (6·7)	19·9 (8·7)
31	Shankaran 2012 [55]¶¶	18,54,0	23,40,0	3328 (557)	3376 (677)	39·1 (1·5)	38·6 (1·6)	NA	NA	6·9 (0·2)	6·9 (0·2)	17·8 (5·9)	18·8 (8·9)
32	Shankaran 2012a [56]¶¶	30,67,0	40,53,0	3391 (620)	3358 (587)	NA	NA	NA	NA	6·9 (0·2)	6·8 (0·2)	18·5 (6·8)	20·5 (8·6)
33	Simbruner 2010 [58] ***	38,24,0	46,17,0	3300 (500)	3300 (600)	39·2 (1·6)	39·4 (1·6)	3·4 (2·4)	3·2 (2·4)	6·9 (0·2)	6·9 (0·2)	19·4 (6·2)	19·5 (4·8)
34	Sun 2012 [59]	6,13,4	7,16,5	3360 (483)	3299 (421)	NA	NA	NA	NA	NA	NA	NA	NA
35	Tanigasalam 2015 [60]	17,42,1	15,41,4	2690 (340)	2760 (297)	39·6 (1·3)	40 (2·4)	NA	NA	6·95 (0·22)	6·93 (0·14)	16·12 (2·93) *	16·18 (0·30)
36	Thayyil 2013 [28]	NA	NA	2977 (402)	2890 (467)	38·0 (1·2)	38·9 (0·8)	NA	NA	NA	NA	NA	NA
37	Thayyil 2021 [10]	41,161,0	39,167,0	2844 (450)	2939 (455)	38·9 (1·3)	39·0 (1·3)	5 (4-6)	5 (4-5)	6·94 (0·25)	6·97 (0·21)	NA	NA
38	Yang 2020 [61]†††	NA	NA	3310 (610)	3410 (600)	38·21 (2·53)	39·10 (2·56)	NA	NA	NA	NA	NA	NA
39	Zhou 2010 [62]	38,41,21	35,41,18	3360 (483)	3299 (421)	NA	NA	NA	NA	NA	NA	NA	NA

Abbreviations

ABG: Arterial blood gas; aEEG: Amplitude-integrated electroencephalography; AS: Apgar score; BD: Base deficit; BE: Base excess; BW: Birth weight; GA: Gestational age; h: hours; HIE: Hypoxic ischemic encephalopathy; g: gram; HR: Heart rate; HT: Hypothermia; min: Minutes; NA: Not available; NE: Neonatal encephalopathy; PPV: Positive pressure ventilation; SD: Standard deviation; TH: therapeutic hypothermia; wk: weeks

*Base excess reported

†Median (IQR)

‡This study reported various outcomes of participants in the Azzopardi 2009 trial. The Azzopardi 2014 study reported clinical outcomes of the participants at the age of 6-7 years. The Roka 2011 study and the Rutherford 2010 study reported MRI outcomes of the participants, enrolled in Hungary and the UK respectively.

§In this trial: TH group had 4 subgroups with cooling to 36·5-36ºC(n=6): 35·9-35·5ºC (n=6): 35±0·5ºC (n=6): 34·5±0·5ºC(n=7). Only those with cooling to 34·5±0·5ºCwere eligible for inclusion in this systematic review, and only their data were extracted.
¶This study is the same as the Battin 2001 trial, however in this study data for TH group included participants cooled to temperature 35·0±0·5 ºC (n=6); or 34·5±0·5 ºC (n=7). Although, the latter conformed to the inclusion criteria of this review outcome data could not be extracted separately for this group· Therefore, data from this study was unusable for meta-analysis.
||Full text not available (relevant information was extracted from the abstract.
[**]This study reported MRI outcomes of participants in the Jacobs 2011trial.
††This trial compared extracorporeal membrane oxygenation (ECMO) with cooling (34º C for 48-72 hours) vs ECMO with normothermia (37ºC)
‡‡Although the inclusion criteria permitted enrolment till 10h of age, the intervention was started within 6 hours in most of the participants (median 4·5 in control group, and median 4 in hypothermia group). Separate data for participants enrolled within 6h was unavailable.
§§Outcomes reported at 18-22 months of age (and not 18-24 months of age).
¶¶Shankaran 2008 reported data on disability at 18 months of participants in the Shankaran 2005 trial; Shankaran 2012reported MRI outcomes of participants in the Shankaran 2005 trial; and Shankaran 2012a reported childhood outcomes of participants in the Shankaran 2005 trial.
||||Although this trial was described as a prospective case control study: randomization was done: hence it was considered a RCT. The trial had 3 arms viz. TH, recombinant erythropoietin, and general supportive care (control group). Data from the TH and control group were analyzed in this systematic review.
[***]Outcomes reported at 18-21 months of age (and not 18-24 months of age).
†††This was a three-arm trial (TH for48 hours group, TH for 72 hours, and normothermia). Data for both TH groups were added for analysis in this review.

Supplementary Table 3. Characteristics of excluded studies

No.	Excluded studies	Reasons for Exclusion	Comments
1	Akula 2015 [64]	Intervention not as per criteria of this systematic review	This was a RCT to determine if temperature regulation is improved during neonatal transport using a servo-regulated cooling device when compared with cooling according to usual center practices. Thus, both the groups received cooling.
2	Azzopardi 2000 [65]	Not a RCT	This was a pilot to study therapeutic hypothermia for neonatal encephalopathy. There was no control arm and all infants received cooling.
3	Azzopardi 2008 [66]	Not a RCT	Study protocol of the Azzopardi 2009 trial
4	Azzopardi 2014 [67]	Outcomes not as per criteria of this systematic review	This study examined the predictive value of aEEG in infants with hypoxic ischemic encephalopathy using data from the Azzopardi 2009 trial.
5	Basu 2016	Outcomes not as per criteria of this systematic review	Post hoc analysis of data from the Gluckman 2005 trial to investigate whether glycemic profile was associated with multiorgan dysfunction and response to hypothermia after perinatal hypoxic-ischaemic encephalopathy.
6	Bonifacio 2011 [69]	Not a RCT	Cohort study of 35 neonates treated with hypothermia and 25 non-treated neonates who underwent magnetic resonance imaging (MRI) to compare association between perinatal events and pattern and extent of brain injury on early MRI.
7	Bonifacio 2012 [70]	Outcomes not as per criteria of this systematic review	Association between hypothermia and MR imaging (quantitative measures) was studied.
8	Catherine 2020a [71]	Outcomes not as per criteria of this systematic review	This study was done to assess whether serum levels of neuronal biomarkers correlated with neurodevelopment outcomes at 18 months. The study reported some data of interest to this review, but they were already extracted from Catherine 2020.
9	Debillon 2003 [72]	Not a RCT	This was a pilot study on 25 term infants to test the practicability and safety of whole body cooling.
10	Eicher 2005a [73]	Timing of outcome measurement not as per the criteria of this systematic review.	Although this review reported outcomes of interest to this review viz. death or disability, and seizures, they were measured at 12 months, and not 18-24 months.
11	Filippi 2012 [74]	Not a RCT	This was a protocol of a pilot study to explore the possible therapeutic role of topiramate in combination with moderate hypothermia.
12	Filippi 2018 [75]	Intervention not as per criteria of this systematic review	This was a multicenter RCT in which participants were randomized to receive either topiramate along with hypothermia or hypothermia alone.
13	Gane 2014 [76]	Timing of outcome measurement not as per the criteria of this systematic review.	Although this review reported outcomes of interest to this review viz. death or disability, disability, and death, they were measured at 12 months, and not 18-24 months.

14	Groenendaal 2013 [77]	Not a RCT	Data were retrieved from an online database to analyze complications and outcomes after implementation of therapeutic hypothermia.
15	Gulliet 2011 [78]	Outcomes not as per criteria of this systematic review.	This study was done to determine whether neurodevelopment outcomes at 18-22 months predicted functional outcomes at 7-8 years among survivors of the Gluckman 2005 trial.
16	Gunn 1998 [79]	Intervention not as per criteria of this systematic review	Participants in the hypothermia group underwent cooling to a rectal temperature of 36.3±0.2°C (n=6), or 35.7±0.2°C (n=6).
17	Gunn 2008 [80]	Outcomes not as per criteria of this systematic review.	This study was a secondary analysis of data from the Gluckman2005 trial, to evaluate whether therapeutic hypothermia altered the prognostic value of clinical grading of neonatal encephalopathy.
18	Horn 2006 [81]	Intervention not as per criteria of this systematic review	This was a RCT in which after cooling the first 4 babies (temp 35-35.5°C), it was clear that repeated revisions to the cooling technique had to be made, which was inappropriate in the context of a RCT. The study was therefore stopped and the data for the 4 cooled infants were presented as a technical report.
19	Jenkins 2012 [82]	Outcomes not as per criteria of this systematic review.	This study was a secondary analysis of RCT (Eicher 2005), to investigate the effects of time and hypothermia on serum cytokine levels.
20	Kwon 2011 [83]	Outcomes not as per criteria of this systematic review.	Secondary analysis of the Shankaran 2005 trial, in which the authors evaluated the association between the presence of clinical neonatal seizures recorded at any time during the hospitalization and the combined outcome of death or moderate or severe disability at 18 months
21	Laptook 2009 [84]	Outcomes not as per criteria of this systematic review.	Secondary analysis of the infants enrolled in the Shankaran 2005 trial to determine whether Apgar scores at 10 minutes were associated with death or disability in early childhood after perinatal hypoxic-ischemic encephalopathy.
22	Laptook 2014 [85]	Not a RCT	This was a retrospective comparison of infants undergoing hypothermia using two different cooling modes of the Blanketrol device.
23	Laptook 2017 [86]	Intervention not as per criteria of this systematic review	In this trial, cooling therapy was initiated at the postnatal age of 6-24 hours.
24	Maoulainine 2017 [87]	Not a RCT	This was a prospective study to assess the feasibility of hypothermia, and outcomes in the newborns, however participants were not randomized.
25	Massaro 2013 [88]	Not a RCT	This was a prospective observational study to determine if biomarkers were elevated in neonates with hypoxic ischemic encephalopathy who died or had severe MRI injury compared with surviving infants with minimal or no injury on brain MRI.
26	Natarajan 2013 [89]	Outcomes not as per criteria of this systematic review	This study examined the association between 10 minute Apgar scores and outcomes at 6-7 years of age in participants enrolled in the Shankaran 2005 trial.

27	Pappas 2015 [90]	Outcomes not as per criteria of this systematic review	This study examined data from school age survivors of the Shankaran 2005 trial to explore predictors of cognitive outcomes.
28	Parikh 2009 [91]	Outcomes not as per criteria of this systematic review	This study examined the relationship between hypothermia treatment and usual care (control) to absolute and relative cerebral tissue volumes, using data from the Shankaran 2005 trial.
29	Persianinov 1978 [92]	Not a RCT	The citation of this paper published in 1978was in Russian. Neither the abstract nor the full text was available. It was deemed highly unlikely that its characteristics would match the criteria for inclusion in this systematic review.
30	Ramiro (Nuñez-Ramiro) 2019 [93]	Intervention not as per criteria of this systematic review	This was a RCT in which both groups received hypothermia, and infants were randomized to either topiramate or placebo.
31	Rivero-Arias 2019 [94]	Outcomes not as per criteria of this systematic review	This study examined data from the Azzopardi 2009 trial in surviving children at 6-7 years of age, to quantify the relationship between overall disability levels and cost of care.
32	Robertson 2011 [95]	Not a RCT	This was a study protocol for a pilot RCT of therapeutic hypothermia, using serial cranial ultrasound, during follow-up of enrolled infants.
33	Rogers 2014 [96]	Intervention not as per criteria of this systematic review	This was a phase 1 clinical trial in which participants received up to 6 doses of erythropoietin in addition to hypothermia.
34	Roka 2007 [97]	Not a RCT	This study examined the safety and technical feasibility of TH, compared against a historical control group.
35	Selway 2010 [98]	Not a RCT	This was a narrative review article
36	Shankaran 2012b [99]	Not a RCT	This was a secondary analysis of participants in the intervention arm of the Shankaran 2005 trial, to examine the target temperature achieved during the intervention.
37	Shankaran 2014 [100][100]	Intervention not as per criteria of this systematic review	This was a RCT in which neonates were assigned to 4 hypothermia groups; 33.5°C for 72 hours, 32.0°C for 72 hours, 33.5°C for 120 hours, or 32.0°C for 120 hours, but there was no arm that received no cooling.
38	Shankaran 2017 [101]	Outcomes not as per criteria of this systematic review	This study examined data from the Shankaran 2005 trial to examine whether perinatal sentinel events were associated with MRI findings linked to death or disability.
39	Shankaran 2017a [102]	Intervention not as per criteria of this systematic review	This was a RCT in which neonates were assigned to 4 hypothermia groups; 33.5°C for 72 hours, 32.0°C for 72 hours, 33.5°C for 120 hours, and 32.0°C for 120 hours, but there was no arm that received no cooling.
40	Thayyil 2017 [103]	Not a RCT	This was the protocol of the Thayyil 2021 trial.
41	Walsh 2013 [104]	Not a RCT	This was a prospective cohort study to determine whether hypothermia alters the discriminative ability of postnatal nucleated red blood cells to distinguish between mild and moderate/severely encephalopathic infants

42	Wusthoff 2011 [105]	Not a RCT	This was a prospective study to determine the incidence and timing of electroencephalographic seizures in term neonates undergoing whole-body therapeutic hypothermia.
43	Wyatt 2007 [106]	Not a RCT	This study was done to evaluate the role of factors that may determine the efficacy of mild systemic hypothermia for neonatal encephalopathy, examining data from the Gluckman 2005 trial.
44	Zhou 2003 [107]	Outcomes not as per criteria of this systematic review	This study was published in Chinese and was translated to English, however the reported outcomes (evaluation of cardiac function and degree of myocardial damage) did not match the criteria for this review.
45	Zonnenberg 2016 [108]	Not a RCT	This was a cohort study that examined the outcomes of patients with perinatal asphyxia who received therapeutic hypothermia, compared to a historical cohort.
46	Zupan 2001 [109]	Not a RCT	This was a multicentric non-randomized trial to assess the safety and efficacy of whole body hypothermia.

Supplementary Table 4. Assessment of risk of bias in the included studies.

**No.**	**Publication**			**Random sequence generation**	**Allocation concealment**	**Selective reporting**	**Blinding (Participants, personnel)**	**Blinding (outcome assessor)**	**Incomplete outcome data**	**Overall risk of bias**
1	Aker 2019 [31]		Reporting	Not reported	SNOSE	Not detected	Not done	Done (for MRI outcomes) Not done (for clinical outcomes)	<10% missing data in either group, for all outcomes	
			Assessment	Unclear (high risk)	Adequate (low risk)	Adequate (low risk)	Inadequate (low risk)*	Adequate (low risk)	Adequate (low risk)	Moderate
2	Akisu 2003 [32]		Reporting	Computer generated	Not reported	Not detected	Not done	Not reported	No missing data for any outcome	
			Assessment	Adequate (low risk)	Unclear (high risk)	Adequate (low risk)	Inadequate (low risk)*	Unclear (high risk)	Adequate (low risk)	High
3	Azzopardi 2009 [30]		Reporting	Secure web based system	By telephone	Not detected	Not done	Done (for neurologic outcomes at 18-24 mo). Not done (for length of hospital stay)	<10% missing data in either group, for all outcomes	
			Assessment	Adequate (low risk)	Adequate (low risk)	Adequate (low risk)	Inadequate (low risk)*	Adequate (low risk)	Adequate (low risk)	Low
4	Azzopardi 2014 [33]		Reporting	Not reported; however, this is a follow-up of participants in the Azzopardi 2009 trial.	Not reported; however, this is a follow-up of participants in the Azzopardi 2009 trial.	Not detected	Not done	Done (for neurologic outcomes at 6-7y).	>10% missing data (17% in TH group & 27% in control group for disability at age >5y; and 16% in HT	

									group & 24% in control group, for cerebral palsy at age>5y	
			Assessment	Adequate (low risk)	Adequate (low risk)	Adequate (low risk)	Inadequate (low risk)*	Adequate (low risk)	Inadequate (high risk)	Moderate
5	Battin 2001 [34]		Reporting	Computer generated	Sealed envelope	Not detected	Not done	Done (for neurologic assessment)	No missing data for any outcome	
			Assessment	Adequate (low risk)	Adequate (low risk)	Adequate (low risk)	Inadequate (low risk)*	Adequate (low risk)	Adequate (low risk)	Low
6	Battin 2003 [63]		Reporting	Computer generated	Sealed envelope	Not detected	Not done	Done (for neurologic assessment)	No missing data for any outcome	
			Assessment	Adequate (low risk)	Adequate (low risk)	Adequate (low risk)	Inadequate (low risk)*	Adequate (low risk)	Adequate (low risk)	Low
7	Bharadwaj 2012 [35]		Reporting	Computer generated	Sealed opaque envelope	Not detected	Not done	Not reported; however the main outcome was mortality before discharge, and length of hospital stay.	<10% missing data in either group, for all outcomes	
			Assessment	Adequate (low risk)	Adequate (low risk)	Adequate (low risk)	Inadequate (low risk)*	Adequate (low risk)	Adequate (low risk)	Low
8	Bhat 2006 [36]		Reporting	Computer generated	Not reported	Not detected	Not done	Not reported	No missing data for the outcomes assessed	
			Assessment	Adequate (low risk)	Unclear	Adequate (low risk)	Inadequate (low risk)*	Unclear	Adequate (low risk)	High
9	Campbell 2018 [37]		Reporting	This is a follow-up study of the Azzopardi 2009 trial	This is a follow-up study of the Azzopardi 2009 trial	Not detected	Not reported	Not reported	Missing data for 35% in TH and 38% in control	
			Assessment	Adequate (low risk)	Adequate (low risk)	Adequate (low risk)	Unclear (moderate risk)	Adequate (low risk)	Inadequate (moderate risk)*	Moderate
10	Catherine 2020 [38]		Reporting	Computer generated	SNOSE	Not detected	Not done	Done (for neurologic assessment) Not done (for duration of hospitalization) Not applicable (for mortality)	<10% missing data in either group, for all outcomes	
			Assessment	Adequate (low risk)	Adequate (low risk)	Adequate (low risk)	Inadequate (low risk)*	Adequate (low risk)	Adequate (low risk)	Low
11	Chen 2018 [39]		Reporting	Not reported	Not reported	Not detected	Not done	Not reported	10% missing data in each group	

			Assessment	Unclear (high risk)	Unclear (high risk)	Adequate (low risk)	Inadequate (low risk)*	Unclear (high risk)	Adequate (low risk)	**High**
12	Cheong 2012 [40]		Reporting	Subgroup analysis of the Jacobs 2011 trial	Subgroup analysis of the Jacobs 2011 trial	Not detected	Not done	Done (for MRI outcomes)	No missing data for the outcomes extracted	
			Assessment	Adequate (low risk)	Adequate (low risk)	Adequate (low risk)	Inadequate (low risk)*	Adequate (low risk)	Adequate (low risk)	Low
13	Eicher 2005 [41]		Reporting	Web based system	Not reported	Not detected	Not done	Not done (for seizures & mortality before discharge)	<10% missing data in either group, for all outcomes	
			Assessment	Adequate (low risk)	Unclear (high risk)	Adequate (low risk)	Inadequate (low risk)	Inadequate (high risk)	Adequate (low risk)	High
14	Field 2013 [42]		Reporting	Web based system	Not reported	Not detected	Not done	Done (for neurologic assessment)	<10% missing data in either group, for all outcomes	
			Assessment	Adequate (low risk)	Unclear (high risk)	Adequate (low risk)	Inadequate (low risk)	Adequate (low risk)	Adequate (low risk)	Moderate
15	Gluckman 2005 [29]		Reporting	Computer generated	SNOSE	Not detected	Not done	Done (for neurologic assessment)	<10% missing data in either group, for all outcomes except disability at ≥ 18 months (10% in each group), and seizures at ≥ 18 months (10% in TH group & 12% in control group)	
			Assessment	Adequate (low risk)	Adequate (low risk)	Adequate (low risk)	Inadequate (low risk)*	Adequate (low risk)	Adequate (low risk)	Low
16	Inder 2004 [43]		Reporting	Not reported	Not reported	Not detected	Not done	Done (for MRI outcomes). Not done seizures)	<10% missing data in either group, for all outcomes	
			Assessment	Unclear (high risk)	Unclear (high risk)	Adequate (low risk)	Inadequate (low risk)*	Adequate (low risk)	Adequate (low risk)	High
17	Jacobs 2011 [26]		Reporting	Computer generated	SNOSE	Not detected	Not done	Done (for neurologic assessment)	<10% missing data in either group, for all outcomes except disability at ≥ 18 months (14% in control group)	

			Assessment	Adequate (low risk)	Adequate (low risk)	Adequate (low risk)	Inadequate (low risk)*	Adequate (low risk)	Adequate (low risk)	Low
18	Jose 2018 [44]		Reporting	Not reported	Not reported	Not detected	Not done	Not reported	>10% missing data in control group	
			Assessment	Unclear (high risk)	Unclear (high risk)	Adequate (low risk)	Inadequate (low risk)*	Unclear (high risk)	Inadequate (high risk)	High
19	Joy 2012 [45]		Reporting	Computer generated	Sequentially numbered, sealed envelopes	Not detected	Not done	Done (for neurologic assessment at discharge)	No missing data in either group	
			Assessment	Adequate (low risk)	Adequate (low risk)	Adequate (low risk)	Inadequate (low risk)*	Adequate (low risk)	Adequate (low risk)	Low
20	Li 2009 [46]		Reporting	Not reported	Not reported	Not detected	Not done	Not reported	>10% missing data (17% in TH group for all outcomes)	
			Assessment	Unclear (high risk)	Unclear (high risk)	Adequate (low risk)	Inadequate (low risk)*	Unclear (high risk)	Inadequate (high risk)	High
21	Lin 2006 [47]		Reporting	Based on odd or even day of admission	Not reported	Not detected	Not done	Not done (but only data on mortality before discharge was extracted)	No missing data	
			Assessment	Inadequate (high risk)	Unclear (high risk)	Adequate (low risk)	Inadequate (low risk)*	Adequate (low risk)	Adequate (low risk)	High
22	Perrone 2010 [48]		Reporting	Internet programme	By switchboard telephone	Not detected	Not done	Outcome reported was mortality	Not applicable (no data were extracted)	
			Assessment	Adequate (low risk)	Adequate (low risk)	Adequate (low risk)	Inadequate (low risk)*	Adequate (low risk)	Not applicable	Low
23	Rakesh 2017 [49]		Reporting	Computer generated	Sealed opaque envelope	Not detected	Not done	Done (for neurologic assessment)	No missing data in either group	
			Assessment	Adequate (low risk)	Adequate (low risk)	Adequate (low risk)	Inadequate (low risk)*	Adequate (low risk)	Adequate (low risk)	Low
24	Robertson 2008 [50]		Reporting	Method not reported, although it was mentioned that it was done by an offsite team	Sealed envelopes	Not detected	Not done	Done (for neurologic assessment)	No drop out for the reported outcomes	
			Assessment	Unclear (moderate risk)	Adequate (low risk)	Adequate (low risk)	Inadequate (low risk)*	Adequate (low risk)	Adequate (low risk)	Moderate
25	Roka 2011 [51]		Reporting	Sub study of the Azzopardi 2009 trial	Sub study of the Azzopardi 2009 trial	Sub study of the Azzopardi 2009 trial	Not done	Not done (for aEEG and MRI data)	>10% missing data (27% in control group)	

			Assessment	Adequate (low risk)	Adequate (low risk)	Adequate (low risk)	Inadequate (low risk)*	Inadequate (high risk)	Inadequate (high risk)	Moderate
26	Rutherford 2010 [52]		Reporting	Subgroup analysis of the Azzopardi 2009 trial	Subgroup analysis of the Azzopardi 2009 trial	Not detected	Not done	Done (for MRI outcomes)	No missing data (for MRI outcomes)	
			Assessment	Adequate (low risk)	Adequate (low risk)	Adequate (low risk)	Inadequate (low risk)*	Adequate (low risk)	Adequate (low risk)	Low
27	Shankaran 2002 [53]		Reporting	Sequence “generated by a random, permuted block algorithm with variable block sizes”, but method of generating the sequence not described.	“Assignments were not masked”	Not detected	Not done	Not reported	No missing data	
			Assessment	Unclear (moderate risk)	Inadequate (high risk)	Adequate (low risk)	Inadequate (low risk)*	High risk	Adequate (low risk)	High
28	Shankaran 2005 [27]		Reporting	Sequence “generated by a random, permuted block algorithm with variable block sizes”, but method of generating the sequence not described.	By telephone	Not detected	Not done	Done (for neurologic assessment)	<10% missing data from either group (for neurologic assessment).	
			Assessment	Unclear (moderate risk)	Adequate (low risk)	Adequate (low risk)	Inadequate (low risk)*	Adequate (low risk)	Adequate (low risk)	Moderate
29	Shankaran 2008 [54]		Reporting	Same as the Shankaran 2005 trial	Same as the Shankaran 2005 trial	Not detected	Not done	Done (for neurologic assessment)	<10% missing data from either group (for neurologic assessment).	
			Assessment	Unclear (moderate risk)	Adequate (low risk)	Adequate (low risk)	Inadequate (low risk)*	Adequate (low risk)	Adequate (low risk)	Moderate
30	Shankaran 2012 [55]		Reporting	Same as the Shankaran 2005 trial	Same as the Shankaran 2005 trial	Not detected	Not done	Not reported (for MRI outcomes)	<10% missing data in either group, for all outcomes	
			Assessment	Unclear (moderate risk)	Adequate (low risk)	Adequate (low risk)	Inadequate (low risk)*	Unclear (high risk)	Adequate (low risk)	Moderate

31	Shankaran 2012a [56]		Reporting	Same as the Shankaran 2005 trial	Same as the Shankaran 2005 trial	Not detected	Not done	Done (for neurologic assessment)	12% missing data in control group in the denominator. 12% missing data in TH group, and 26% in control group for disability at age >5y; and 12% missing data in TH group, and 24% in control group for cerebral palsy	
			Assessment	Unclear (moderate risk)	Adequate (low risk)	Adequate (low risk)	Inadequate (low risk)*	Adequate (low risk)	Inadequate (high risk)	High
32	Shimi 2014 [57]		Reporting	Not reported	Closed envelope	Not detected	Not done	Not reported	No missing data for the reported outcomes	
			Assessment	Unclear	Adequate (low risk)	Adequate (low risk)	Inadequate (low risk)*	Unclear	Adequate(low risk)	High
33	Simbruner 2010 [58]		Reporting	Not reported	Sealed envelopes	Not detected	Not done	Done (for neurologic assessment)	>10% missing data in both the groups (17% in TH group, and 11% in control group)	
			Assessment	Unclear	Adequate (low risk)	Adequate (low risk)	Inadequate (low risk)*	Low risk	Inadequate (high risk)	Moderate
34	Sun 2012 [59]		Reporting	Computer generated	SNOSE	Not detected	Not done	Not reported	No missing data for the reported outcomes	
			Assessment	Adequate (low risk)	Adequate (low risk)	Adequate (low risk)	Inadequate (low risk)*	Unclear	Adequate (low risk)	Moderate
35	Tanigasalam 2015 [60]		Reporting	Computer generated	Sealed opaque envelopes	Not detected	Not done	Not reported	No missing data for the reported outcomes	
			Assessment	Adequate (low risk)	Adequate (low risk)	Adequate (low risk)	Inadequate (low risk)*	Unclear	Adequate (low risk)	Moderate
36	Thayyil 2013 [28]		Reporting	Computer programme	Not reported	Not detected	Not done	Not reported	No missing data for the reported outcomes	
			Assessment	Adequate (low risk)	Unclear	Adequate (low risk)	Inadequate (low risk)*	Unclear	Adequate (low risk)	High
37	Thayyil 2021 [10]		Reporting	Internet programme	Sealed envelope	Not detected	Not done	Done (for neurologic and MRI assessments).	<10% missing data in either group, for	

									all outcomes at 18-24 months	
			Assessment	Adequate (low risk)	Adequate (low risk)	Adequate (low risk)	Inadequate (low risk)*	Adequate (low risk)	Adequate (low risk)	Low
38	Yang 2020 [61]		Reporting	Computer generated	Not reported	Not detected	Not done	Not applicable to "mortality before discharge"	No missing data	
			Assessment	Adequate (low risk)	Unclear	Adequate (low risk)	Inadequate (low risk)*	Adequate (low risk)	Adequate (low risk)	High
39	Zhou 2010 [62]		Reporting	Computer generated codes	SNOSE	Not detected	Not done	Done (for neurologic assessment at 18-24 mo)	>10% missing data (27% missing data in TH group and 20% in control group, for mortality & mortality or disability at 18-24 mo; and 32% in TH group & 26% in control group, for cerebral palsy at 18-24 months.	
			Assessment	Adequate (low risk)	Adequate (low risk)	Adequate (low risk)	Inadequate (low risk)*	Adequate (low risk)	Inadequate (high rosk)	Moderate

Abbreviations: aEEG: amplitude integrated electroencephalogram; MRI: magnetic resonance imaging; SNOSE: Serially numbered, Opaque, sealed envelopes; TH: therapeutic hypothermia

*The nature of the trial was such that lack of blinding of participants (neonates with encephalopathy) was not a source of bias. Similarly, the outcomes evaluated were such that lack of blinding of family members of enrolled neonates, was not a source of bias.

Supplementary Table 5: PRISMA 2020 checklist

Section and Topic	Item #	Checklist item	Location where item is reported
**TITLE**			
Title	1	Identify the report as a systematic review.	Title page
**ABSTRACT**			
Abstract	2	See the PRISMA 2020 for Abstracts checklist.	Line 2-44
**INTRODUCTION**			
Rationale	3	Describe the rationale for the review in the context of existing knowledge.	Lines 75-86
Objectives	4	Provide an explicit statement of the objective(s) or question(s) the review addresses.	Lines 81-86
**METHODS**			
Eligibility criteria	5	Specify the inclusion and exclusion criteria for the review and how studies were grouped for the syntheses.	Lines 92-122
Information sources	6	Specify all databases, registers, websites, organisations, reference lists and other sources searched or consulted to identify studies. Specify the date when each source was last searched or consulted.	Lines 124-131
Search strategy	7	Present the full search strategies for all databases, registers and websites, including any filters and limits used.	Lines 132-137<br>Suppl Table 1
Selection process	8	Specify the methods used to decide whether a study met the inclusion criteria of the review, including how many reviewers screened each record and each report retrieved, whether they worked independently, and if applicable, details of automation tools used in the process.	Lines 138-144
Data collection process	9	Specify the methods used to collect data from reports, including how many reviewers collected data from each report, whether they worked independently, any processes for obtaining or confirming data from study investigators, and if applicable, details of automation tools used in the process.	Lines 147-161
Data items	10a	List and define all outcomes for which data were sought. Specify whether all results that were compatible with each outcome domain in each study were sought (e.g. for all measures, time points, analyses), and if not, the methods used to decide which results to collect.	Lines 113-122
	10b	List and define all other variables for which data were sought (e.g. participant and intervention characteristics, funding sources). Describe any assumptions made about any missing or unclear information.	Lines 147-159
Study risk of bias assessment	11	Specify the methods used to assess risk of bias in the included studies, including details of the tool(s) used, how many reviewers assessed each study and whether they worked independently, and if applicable, details of automation tools used in the process.	Lines 172-175
Effect measures	12	Specify for each outcome the effect measure(s) (e.g. risk ratio, mean difference) used in the synthesis or presentation of results.	Lines 164-170
Synthesis	13a	Describe the processes used to decide which studies were eligible for each synthesis (e.g. tabulating the	Line 147-159

Section and Topic	Item #	Checklist item	Location where item is reported
methods		study intervention characteristics and comparing against the planned groups for each synthesis (item #5)).	
	13b	Describe any methods required to prepare the data for presentation or synthesis, such as handling of missing summary statistics, or data conversions.	Lines 1601-161
	13c	Describe any methods used to tabulate or visually display results of individual studies and syntheses.	Lines 162-171
	13d	Describe any methods used to synthesize results and provide a rationale for the choice(s). If meta-analysis was performed, describe the model(s), method(s) to identify the presence and extent of statistical heterogeneity, and software package(s) used.	Lines 164-171
	13e	Describe any methods used to explore possible causes of heterogeneity among study results (e.g. subgroup analysis, meta-regression).	Lines 172-175
	13f	Describe any sensitivity analyses conducted to assess robustness of the synthesized results.	Lines 188-189
Reporting bias assessment	14	Describe any methods used to assess risk of bias due to missing results in a synthesis (arising from reporting biases).	Mentioned in the Results section at appropriate places
Certainty assessment	15	Describe any methods used to assess certainty (or confidence) in the body of evidence for an outcome.	Lines 171-173
**RESULTS**			
Study selection	16a	Describe the results of the search and selection process, from the number of records identified in the search to the number of studies included in the review, ideally using a flow diagram.	Lines 192-198<br>Fig 1
	16b	Cite studies that might appear to meet the inclusion criteria, but which were excluded, and explain why they were excluded.	Line 195-196<br>Supplementary table 3
Study characteristics	17	Cite each included study and present its characteristics.	Table 1<br>Supplementary Table 2
Risk of bias in studies	18	Present assessments of risk of bias for each included study.	Summarized in Results and Supplementary Table 4
Results of individual studies	19	For all outcomes, present, for each study: (a) summary statistics for each group (where appropriate) and (b) an effect estimate and its precision (e.g. confidence/credible interval), ideally using structured tables or plots.	Lines 213-330<br>Tables 2,3,4,<br>Fig 2-16
Results of syntheses	20a	For each synthesis, briefly summarise the characteristics and risk of bias among contributing studies.	Supplementary Table 4
	20b	Present results of all statistical syntheses conducted. If meta-analysis was done, present for each the summary estimate and its precision (e.g. confidence/credible interval) and measures of statistical heterogeneity. If comparing groups, describe the direction of the effect.	Lines 213-330<br>Tables 2,3,4,<br>Fig 2-16
	20c	Present results of all investigations of possible causes of heterogeneity among study results.	Mentioned at appropriate places in Results
	20d	Present results of all sensitivity analyses conducted to assess the robustness of the synthesized results.	Lines 345-355

Section and Topic	Item #	Checklist item	Location where item is reported
			Table 4
Reporting biases	21	Present assessments of risk of bias due to missing results (arising from reporting biases) for each synthesis assessed.	Presented at appropriate places in Results
Certainty of evidence	22	Present assessments of certainty (or confidence) in the body of evidence for each outcome assessed.	Not presented separately
**DISCUSSION**			
Discussion	23a	Provide a general interpretation of the results in the context of other evidence.	Lines 357-371
	23b	Discuss any limitations of the evidence included in the review.	Presented at multiple places in Results
	23c	Discuss any limitations of the review processes used.	Lines 392-421
	23d	Discuss implications of the results for practice, policy, and future research.	Lines 424-430
**OTHER INFORMATION**			
Registration and protocol	24a	Provide registration information for the review, including register name and registration number, or state that the review was not registered.	Lines 507-510
	24b	Indicate where the review protocol can be accessed, or state that a protocol was not prepared.	Lines 510
	24c	Describe and explain any amendments to information provided at registration or in the protocol.	Lines 390-391
Support	25	Describe sources of financial or non-financial support for the review, and the role of the funders or sponsors in the review.	No funding (mentioned in Title page)
Competing interests	26	Declare any competing interests of review authors.	No interests.<br>(mentioned in Title page
Availability of data, code and other materials	27	Report which of the following are publicly available and where they can be found: template data collection forms; data extracted from included studies; data used for all analyses; analytic code; any other materials used in the review.	Mentioned in Title page
